# Synthesis of Nano/Microsized MIL-101Cr Through Combination of Microwave Heating and Emulsion Technology for Mixed-Matrix Membranes

**DOI:** 10.3389/fchem.2019.00777

**Published:** 2019-11-19

**Authors:** Irina Gruber, Alexander Nuhnen, Arne Lerch, Sandra Nießing, Maximilian Klopotowski, Annika Herbst, Matthias Karg, Christoph Janiak

**Affiliations:** ^1^Institut für Anorganische Chemie und Strukturchemie, Heinrich-Heine-Universität Düsseldorf, Düsseldorf, Germany; ^2^Institut für Physikalische Chemie, Heinrich-Heine-Universität Düsseldorf, Düsseldorf, Germany

**Keywords:** metal-organic framework (MOF), MIL-101Cr, nano/microsized MOF, microwave heating, emulsion, surfactants, mixed-matrix membranes (MMMs)

## Abstract

Nano/microsized MIL-101Cr was synthesized by microwave heating of emulsions for the use as a composite with Matrimid mixed-matrix membranes (MMM) to enhance the performance of a mixed-gas-separation. As an example, we chose CO_2_/CH_4_ separation. Although the incorporation of MIL-101Cr in MMMs is well-known, the impact of nanosized MIL-101Cr in MMMs is new and shows an improvement compared to microsized MIL-101Cr under the same conditions and mixed-gas permeation. In order to reproducibly obtain nanoMIL-101Cr microwave heating was supplemented by carrying out the reaction of chromium nitrate and 1,4-benzenedicarboxylic acid in heptane-in-water emulsions with the anionic surfactant sodium oleate as emulsifier. The use of this emulsion with the phase inversion temperature (PIT) method offered controlled nucleation and growth of nanoMIL-101 particles to an average size of <100 nm within 70 min offering high apparent BET surface areas (2,900 m^2^ g^−1^) and yields of 45%. Concerning the CO_2_/CH_4_ separation, the best result was obtained with 24 wt.% of nanoMIL-101Cr@Matrimid, leading to 32 Barrer in CO_2_ permeability compared to six Barrer for the neat Matrimid polymer membrane and 21 Barrer for the maximum possible 20 wt.% of microMIL-101Cr@Matrimid. The nanosized filler allowed reaching a higher loading where the permeability significantly increased above the predictions from Maxwell and free-fractional-volume modeling. These improvements for MMMs based on nanosized MIL-101Cr are promising for other gas separations.

## Introduction

Starting at the beginning of the 1990s metal-organic frameworks (MOFs) as new porous materials have been constructed from metal-atom or metal-cluster building blocks and bridging organic linkers (Batten and Robson, 1998). It became possible to produce extended anionic, cationic and neutral porous frameworks with unique pore architectures and functions (Yaghi et al., 1998). However, porosity was to some extent counter-acted when independent one-, two-, and even three-dimensional nets interpenetrated into each other in many solid-state structures of polymeric, hydrogen-bonded nets, and coordination polymers (Batten and Robson, 1998). The existence of building units with different sizes, yet close topologies, resulted into “scalable or isoreticular chemistry” giving predictable frameworks (Férey, 2000). Secondary building units are the metal or cluster entities with their directly coordinating ligand groups. Consideration of the geometric and chemical attributes of the secondary building units and linkers leads to prediction of the framework topology (Eddaoudi et al., 2001). It became quickly apparent, how molecular complexes and clusters can be transformed to extended solids (Yaghi et al., 2000). For chemical and physical functionalities robustness of a metal-organic framework is required in the absence of guest molecules in the cavities. The stability depends mainly on the structural dimensionality of the networks (Kitagawa and Kondo, 1998). MOFs quickly emerged as a competitively investigated class of porous materials (Yaghi et al., 2003). MOFs promise applications in small molecule adsorption and storage (Li et al., 2009), gas and liquid separation (Tanh Jeazet et al., 2012), catalysis (Beyzavi et al., 2015), sensing (Chen et al., 2009), drug delivery (Horcajada et al., 2012), heat transformation, and other applications (Janiak and Henninger, 2013; Azar and Keskin, 2018; Erucar and Keskin, 2018). However, the inherent microporosity of most metal-organic frameworks prevents a fast access of molecules to the internal surface in larger MOF crystallites. A faster access of molecules to the internal surface is desirable for application in gas adsorption/desorption and storage and can be achieved by reduction of the diffusion path length by decreasing the crystal size (Rieter et al., 2006; Lin et al., 2009) or by enlargement of the pore dimensions through defects toward hierarchical micro-meso-macropores (Shen et al., 2015). In this work, we focus on the reduction of the diffusion path length through decrease of the particle size. There are various approaches to control the MOF particle size, for example, microwave-assisted routes (Khan et al., 2011), surfactant-mediated syntheses (Huang et al., 2003), reverse microemulsions (Lin et al., 2009), and sonochemistry (Khan and Jhung, 2015). Among these strategies microwave heating and the use of surfactants have been the most advantageous methods (Khan and Jhung, 2015; Huang et al., 2018). Microwave heating is known to enhance rates of nucleation and crystal growth processes, including acceleration of nucleation over crystal growth (Diring et al., 2010). Moreover, in microwave reactions the required temperatures can be reached within seconds (Galema, 1997) and microwave heating is an “instant on/instant off” energy source, significantly reducing the risk of overheating reactions (Bogdal, 2006).

**Figure 1:**
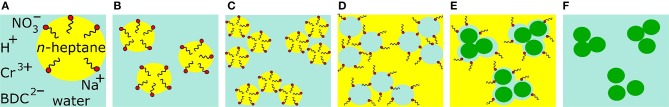
The anticipated direct emulsion mechanism for the formation of nanoMIL-101Cr (depicted as green spheres). BDC^2−^, benzene-1,4-dicarboxylate; Cr^3+^, chromium salt; ${\text{NO}}_{3}^{-}$, nitrate ion; and micelles with hydrophobic groups of sodium oleate oriented toward the center of heptane oil droplets (hydrophilic groups = red spheres). MOF precursors are in the aqueous phase **(A)**. At higher temperature, the heptane droplets expand rapidly **(B)**, at the PIT (phase inversion temperature) the size of the heptane droplet reaches a minimum **(C)**, phase inversion occurs **(D)**, reverse micelles work as nanoreactors for nanoMIL-101Cr and in addition coalescence occurs **(E)**, isolated and agglomerated nanoMIL-101Cr particles in water after washing procedure **(F)**.

Most critical was the emulsion technique, through which we tried to achieve two goals: The first one was the reduction of the particle size, the second goal was good yields. As shown in Table S1, already published results for MIL-101Cr via microwave heating show particle sizes between 49 and 200 nm. A view on the described yields shows relatively low yields (35-38%) (Khan et al., 2011) or no statement concerning the yield (Wuttke et al., 2015). Emulsions stabilized by surfactants also help to control MOF nucleation and growth through micelles, which can work as nanoreactors (Khan et al., 2010). Emulsions can be differentiated between macroemulsions (= emulsions) and microemulsions (Wu et al., 2017). Macroemulsions are built of dispersed droplets with radii in the range of 1-90 μm, whereas microemulsions are built of dispersed droplets with radii in the range of 5–50 nm (Carr and Shantz, 2005). Both can be further differentiated in direct and reverse emulsions (Figure 1, Figure S6). Direct emulsions are given from hydrophobic “oil” droplets dispersed in a hydrophilic medium (water) (Salvia-Trujillo et al., 2018), whereas reverse emulsions are formed from water droplets dispersed in a hydrophobic medium (organic solvent, oil). The size of the droplets or micelles depends strongly on the water to surfactant ratio and on the temperature. If a high reaction temperature is required, coalescence of the micelles will occur and especially microemulsions do not remain intact at high temperature (Whitby et al., 2012). Therefore, we speak only generally of emulsions here and do not use the term microemulsion, because droplet sizes vary during the reaction procedure. So far, only reverse microemulsions have been used for the synthesis of MOFs (Rieter et al., 2006) wherein two-micellar systems (different micelles contained either the metal salt or the organic linker) were mixed and the reaction was started through coalescence (Wang et al., 2018). The reaction temperature in reverse microemulsion synthesis for MOFs was between 0 and 120°C (Taylor et al., 2008). There are no reverse emulsion syntheses for MOFs known, where higher temperatures (>180°C) were used.

Two processes are known for generating emulsions with small droplets. The first one is based on high-energy emulsification methods (e.g., ultrasound generators), whereas the second process includes low-energy emulsification methods. Among low energy methods, we can find the most widely used phase inversion temperature (PIT) method which was first described by Shinoda and Saito (1969). Phase inversion of emulsions means the conversion of oil-in-water to water-in-oil system (or vice versa) as shown in Figure 1 and Figure S6. The PIT method is based on the temperature at which the phase transition occurs, such that for example a low temperature favors oil-in-water emulsions and a high temperature favors water-in-oil emulsions (Kale and Deore, 2017). This method leads to a reduction of the interfacial tension of the surfactant, providing droplet fragmentation and inverting the water-in-oil emulsion phase to an oil-in-water emulsion. At the PIT the droplet size and the interfacial tension reaches a minimum. Important information about phase structures in the phase inversion temperature (PIT) range were presented by Benton et al. (1986). Other examples of inversion in the PIT range are “abnormal emulsions.” Here, the surfactants are preferentially located in the dispersed phase of an emulsion (Sajjadi et al., 2004). The PIT depends on surfactant concentration (Izquierdo et al., 2002) as well as on the type of oil and the hydrophilic chain length of the surfactants. Therefore, in the case of an oil-in-water emulsion, the stability increases significantly with the chain length of the hydrophile–lipophile group of the surfactant and the emulsion droplets are usually negatively charged because of the selective adsorption of OH^−^ onto the droplet surfaces (Mei et al., 2011). The PIT method is mostly used in combination with non-ionic surfactants but recent studies have shown that a specific oil emulsion can be produced by using combinations of sodium caseinate and Tween 20 (Su and Zhong, 2016). Also, a non-ionic/anionic surfactant blend exhibits a higher PIT than the corresponding non-ionic-only system. This result can be attributed to the hydrophilic nature of anionic surfactants. Kundu et al. (2013) studied the effect on PIT of an oil-in-water emulsion stabilized by anionic surfactant. Moreover, Kale and Deore (2017) also showed the influence on PIT of an oil-in-water emulsions stabilized by an anionic surfactant. Studies also have shown that the formation and properties of emulsions prepared according to the PIT approach can be modulated by using a combination of non-ionic and cationic surfactants. With increasing cationic surfactant concentration not only the PIT increased but also the positive charge on the oil droplets after emulsion formation (Mei et al., 2011). Positively charged oil-in-water emulsions were prepared by Sun et al. by adding a cationic surfactant to the system. The cationic molecules raise the PIT above 100°C (Mei et al., 2011).

One of the most interesting applications of MOFs is their use in mixed-matrix membranes (MMM) (Sorribas et al., 2014). Pure organic polymer membranes are already applied for gas separation processes in industry due to their cost and energy effectiveness, environmentally friendly use, as well as their simple and versatile manufacturing (Seoane et al., 2015). They are used for CO_2_ removal from natural gas (natural gas sweetening), hydrogen isolation and recovery, and oxygen and nitrogen enrichment from air (Miricioiu et al., 2019). Membrane technology is becoming more important for CO_2_ separation from natural gas as conventional CO_2_ absorption and adsorption processes are generally more energy demanding and costly. Pure polymer membranes are the currently used membranes CO_2_ separation from natural gas. Because of their low permeability and selectivity, plasticization effects, and low thermal and chemical stability inorganic membranes are seen as the future. The latter feature higher permeability, better selectivity, thermal, and chemical stability. Also, permeability and selectivity of neat polymer membranes are inversely correlated to each other such that a rise in permeability usually results in a loss in selectivity and vice versa. The current maximum selectivity for a given permeability is known as the Robeson upper bound limit (Robeson, 1991, 2008). One possibility to overcome the Robeson upper bound is seen in combining the organic polymer with porous filler particles (e.g., MOFs, zeolites) as an additive in so-called mixed-matrix membranes (Dechnik et al., 2017a). When it comes to mixed-matrix membranes for CO_2_/CH_4_ separation the choices geared toward application appear to be (functionalized) zeolite/polymer or zeolite/carbon composites (Yeo et al., 2012). Zeolites can be incorporated into membranes but show poor compatibility with organic polymers. Here the MOFs come into play, because they offer some very interesting opportunities in combination with polymers (Liu et al., 2018). Latest advances in MMM research use MOFs as fillers in the polymer matrix based on their well-defined and tuneable nanoporosity (Zornoza et al., 2013). State-of-the art MOF-based MMM work investigates the design and influence of the surface chemistry and texture, particle size, and morphology, and dispersion of the MOF filler particles on the separation properties of the MMMs. For example, particles smaller than <50 nm in size with their defined shape and orientation and their homogeneity known in the membrane are studied to probe the separation properties. Also, effects on polymer rigidity or free volume are quantified and related to changes in permeability. The MOF-MMM work uses also advanced characterization techniques such as tomographic SEM/TEM to better understand the filler-matrix interactions (Dechnik et al., 2017a). For further information on the state-of-the-art on MOF@polymer MMM work the reader is referred to review articles (Tanh Jeazet et al., 2012; Zornoza et al., 2013; Seoane et al., 2015; Dechnik et al., 2017a,c, Amooghin et al., 2019). One of the most investigated polymers for MMMs is Matrimid 5218 (Figure S3) which shows high mechanical stability, high chemical resistance, and a good permselectivity (Nuhnen et al., 2018). A trend for the next-generation MMMs follows the incorporation of nanosized fillers (Carreon et al., 2017), as it is supposed that the quality of MMMs increases, due to enhanced distribution, less agglomeration, and reduced sedimentation of nanosized filler particles (Zornoza et al., 2011).

For use in membrane technology MOFs with high water stability are necessary and MIL-101Cr (Figure S4) meets the requirement (Janiak and Henninger, 2013). With a view on CO_2_/CH_4_ separation, the adsorption of CO_2_ in MIL-101 is higher than that of CH_4_, because of the higher polarization and quadrupole moment of CO_2_ (Chowdhury et al., 2012). Moreover, Zhao et al. have achieved with nanosized MIL-101Cr a significant enhancement of CO_2_ adsorption compared to bulk MIL-101 due to the “nano-effect” of porous materials for gas adsorption (Zhao et al., 2018). Hence, MIL-101Cr is an appropriate filler in MMMs for CO_2_/CH_4_ gas separation. To the best of our knowledge, there is no study on the nanoMIL-101Cr in Matrimid (nanoMIL-101Cr@Matrimid) for CO_2_/CH_4_ mixed-gas separation. In the present work, the formation of nanoMIL-101Cr (corresponds to MOFs with a size in the nano- and lower micrometer range) was achieved by the combined approach of surfactant emulsion and ultrasonication followed by microwave heating. MicroMIL-101Cr (corresponds to MOFs with a size in the middle micrometer range) was synthesized according to the literature (Yang et al., 2010). Subsequently, we show an improved mixed-gas permeation performance of nano- over microMIL-101Cr@Matrimid membranes toward the separation of the binary gas mixture of CO_2_ and CH_4_.

## Materials and Methods

### Materials

All chemicals were obtained commercially and were used without further purification: Cr(NO_3_)_3_·9H_2_O (Acros Organics, 99%), HNO_3_ (Grüssing, 65 wt.%), 1,4 benzenedicarboxylic acid (H_2_BDC, Acros Organics, >99%), tetramethylammoniumhydroxid (TMAOH, 25 wt.% in water, Sigma Aldrich), sodium oleate (Tokyo Chemical Industry, >97%), hexadecyltrimethylammonium bromide (CTAB, Sigma Aldrich, 95%), Triton X-45 (Sigma Aldrich), Matrimid® 5218 powder (Huntsman, Figure S3), n-heptane (Sigma Aldrich, p.a.), DMF (VWR, p.a.), ethanol (VWR, p.a.), and dichloromethane (DCM, Fisher Chemical, 99.9%). All experimental work was performed in air. De-ionized (DI) water was used.

### Instrumentation

**Powder X-ray diffraction (PXRD)** patterns were obtained on a Bruker D2 Phaser powder diffractometer equipped with a flat silicon, low background sample holder using Cu–K_α_ radiation (λ = 1.5418 Å, 30 kV, 10 Ma, ambient temperature). With this sample holder at 2θ < ~10° the beam spot is strongly broadened so that only a fraction of the reflected radiation reaches the detector; hence lower relative intensities are measured in this range. For all samples 2θ angles between 5 and 50° over a time of 105 min, that is 0.007°/sec, were measured. Simulated patterns of MIL-101Cr were calculated with CCDC Mercury 3.9 program using the single crystal data of MIL-101Cr obtained by Rietveld refinement (CCDC no. 605510, Refcode OCUNAC; Férey et al., 2005). Nitrogen physisorption (N_2_) isotherms were obtained with a Nova 4000e from Quantachrome at 77 K. For measuring the isotherms, the MOF powders were loaded into glass tubes and weighted before they were degassed at 150°C for 2 h under vacuum. After that, the glass tubes were weighted again. At last, they were transferred to the analysis port of the sorption analyzer. Apparent Brunauer-Emmett-Teller (BET) surface areas were calculated from the adsorption branch of the nitrogen physisorption isotherms. In this work we refer to the microporous MOF surface areas from Type I isotherms as “apparent BET” based on reference (Thommes et al., 2015), where it is noted that ‘the BET-area derived from a Type I isotherm must not be treated as a realistic probe accessible surface area' but ‘represents an apparent surface area, which may be regarded as a useful adsorbent “fingerprint.”' Non-linear (NL-)DFT calculations for pore size distribution curves were done with the native NovaWin 11.03 software using the “N_2_ at 77 K on carbon, slit pore, non-linear density functional theory (NLDFT) equilibrium” model (Gelb et al., 1999). Note that the NL-DFT calculations only yield pore size distributions in the micropore (<2 nm, 20 Å) to low mesopore (<10 nm, 100 Å) regime. BET surface areas and pore volumes (measured at P·${\text{P}}_{0}^{-1}$ = 0.95) were calculated from N_2_ sorption isotherms. Infrared (IR) spectra were obtained with a Bruker FT-IR Tensor 37 in attenuated total reflection (ATR) mode using a diamond crystal in the range 4,000–500 cm^−1^. Scanning electron microscopic (SEM) images were recorded with a Zeiss DSM 982 and a Jeol JSM-6510LV QSEM Advanced electron microscope with a LAB-6 cathode at 20 keV. The microscope was equipped with a Bruker Xflash 410 silicon drift detector and the Bruker ESPRIT software for EDX analysis. The membrane cross-sections were prepared through freeze-fracturing after immersion in liquid nitrogen. The membrane fractions were mounted on a sample holder (Figure S1) and coated with gold by a Jeol JFC 1200 fine-coater at an approximate current of 20 mA for 20–30 s. Dark and light areas around cross-section of membranes depend on mounting on SEM sample holder. Light areas are a consequence of the nearby metal sheet which for stability purpose is screwed close to the membrane (Figure S1). Transmission electron microscopy (TEM) micrographs were taken at room temperature with a Zeiss E902 ATEM. Samples were deposited on 200 μm carbon-coated copper grids. The size distribution was calculated from a manual diameter determination over a minimum of 50 isolated particles. Dynamic light scattering (DLS) experiments were conducted with a 3D LS Spectrometer operated in 2D mode (LS Instruments, Fribourg, Switzerland) using a HeNe-laser (λ = 632.8 nm) with a maximum constant output power of 35 mW as light source. The measurements were performed at scattering angles from θ = 30–140° and at a constant temperature of 25°C, which was achieved by a heat-controlled decalin bath connected to a circulating water bath (Julabo CF31). The light scattered by the sample was detected by two avalanche photodetectors operating in pseudo-cross-correlation mode. For all measurements dust-free, disposable culture tubes made of borosilicate glass (Fisher Scientific, Schwerte, Germany) were used. Samples for light scattering experiments were prepared by redispersion of freeze-dried particles in water using ultrasonication and filtration with a syringe filter (5 μm pore size, PTFE, hydrophobic). Temperature stability was achieved by equilibration of the samples for at least 30 min in the decalin bath before the measurements. All measurements were repeated three times using acquisition times of 60 s each. The resulting intensity-time autocorrelation functions were analyzed by the CONTIN algorithm using the software AfterALV 1.0e (Dullware, Wageningen, the Netherlands) yielding the relaxation rate distributions G(Γ). For an angle dependent diffusion analysis the mean relaxation rates Γ of the dominating species in the intensity-weighted G(Γ) were plotted vs. the squared scattering vector *q*^2^ to analyze whether translational diffusion is probed. The polydispersity index (PDI) is given in the figure captions in Supplementary Material [calculated according (Kozlov et al., 2017), PDI = (standard deviation/average nanoparticle size)^2^]. As a **microwave reactor** a CEM Mars 6 with 55 mL sample tubes (2.1 cm diameter, height 19 cm) was used. Microwave heating was done by pre-setting the desired temperature, maximum power (here 600 W), time ramp to reach the desired temperature and hold time before turning off the microwave heating. During ramping and holding the heating is not continuous but supplied by pulses. A **Sonics vibra-cell** VCX 750 ultrasound generator with Microtip 630-0419, Amplitude 20%) was employed for both emulsion types.

### Mixed Gas Permeation

The membranes were placed inside the membrane module composed of two stainless steel parts with a cavity (4.5 cm in diameter) in which a macroporous disk support (20 μm nominal pore size, Mott Corp.) is gripped inside with Viton o-rings. Before the measurement, the membranes were heated for 1 h in a vacuum oven at 150°C for removing residual moisture traces through storage. The composition of the feed gas mixtures and the purge gas flow (sweep gas) were controlled by an **OSMO Inspector** device (Convergence Industry B.V; Figure S2). Gas concentrations in the outgoing stream were evaluated by an Agilent 490 μGC on-line **gas micro-chromatograph** equipped with a thermal conductivity detector (TCD). Gas concentrations in the permeate were measured every 30 min until steady state was reached (up to 8 h). Permeability results in Barrer (1 Barrer = 1 × 10^−10^ cm^3^ (STP)cm/(cm^2^ s cmHg) were obtained from the concentrations in steady state. The real separation selectivity of the mixtures was calculated as the ratio of the mole fractions of the components in the permeate and the feed stream. Permeation measurements were performed at 25°C controlled by a flexible heating coil. The carrier gas was Helium. After the mixed-gas permeation investigations, the thickness of the membranes was measured at 10 different places using a micrometer screw as a **thickness measuring device**. A binary mixture of CO_2_ and CH_4_ (50 vol% of CO_2_) was used as a feed gas in permeability and selectivity measurements. We note that the natural gas composition is not a 1:1 ratio of CO_2_/CH_4_. But as the CO_2_ in natural gas resources varies from 4 to 50% according to Datta and Sen (2006) it is a good approximation to use a 1:1 (v:v) ratio of CO_2_/CH_4_. Furthermore, a review from Yeo et al. (2012) shows that most of the studies of membrane technologies regarding CO_2_ removal from natural gas are done with feed compositions of equimolar ratios of CO_2_/CH_4_. There are also other contaminants in natural gas (Faramawy et al., 2016) but they are hardly ever included in CO_2_/CH_4_ separation studies with MOF@polymer MMMs as such measurements are beyond the scope of the often more fundamental CO_2_/CH_4_ permselectivity studies.

Permeation experiments were conducted at 25°C, 4 bar feed pressure and 1 bar permeate pressure. The permeability of the membranes was calculated by the following two equations (Abedini et al., 2014):

$$\begin{array}{l} P_{CH_{4}}=\frac{2.73.15\;\times 10^{10}\;\left(1-y_{CO_{2}}\right)\;VL}{760\;AT\;\left[\left(1-\;x_{CO_{2}}\right)\left(P_{0}\times 76\right)/14.7\right]}\left(\frac{dP}{dt}\;\right)\\ P_{CO_{2}}=\frac{2.73.15\;\times 10^{10}y_{CO_{2}}VL}{760\;AT\;\left[\;x_{CO_{2}}\left(P_{0}\times 76\right)/14.7\right]}\left(\frac{dP}{dt}\;\right) \end{array}$$

P_CH4_ and P_CO2_ (Barrer): permeability of gases in Barrer;

L (cm): membrane thickness;

T (°C): experiment temperature;

V (cm^3^): constant volume vessel;

A (cm^2^): membrane surface area;

P_o_ (cmHg): feed pressure;

(dP/dt): slope of pressure vs. time.

The selectivity was calculated by the following equation:

$$\begin{array}{l} \alpha_{A/B}=\;\frac{y_{A}/y_{B}}{x_{A}/x_{B}} \end{array}$$

x: mole fractions in the feed gas; y: mole fractions in the permeate.

### Maxwell Model and Bruggeman Model

The Maxwell model can be used to describe the gas transport through a dense composite membrane (Bouma et al., 1997).

For spherical particles the permeability of the composite membrane *P*_*eff*_ can be calculated as follows:

$$\begin{array}{l} P_{eff}=\;P_{c}\;\frac{\;P_{d}\cdot \;\left(1+2\phi_{d}\right)+P_{c}\cdot \;\left(2-2\phi_{d}\right)\;}{P_{d}\cdot \;\left(1-\phi_{d}\right)+P_{c}\cdot \;\left(2+\phi_{d}\right)} \end{array}$$

With *P*_*c*_ as permeability of the continuous phase, *P*_*d*_ is the dispersed phase permeability and ϕ_*d*_ is the volume fraction of the filler.

In cases where the permeability of the filler is much higher than the permeability of the continuous phase (*P*_*d*_ >> *P*_*c*_) the upper equation can be written as follows:

$$\begin{array}{l} P_{d}\gg \;P_{c};\;\frac{P_{eff}}{P_{c}}=\frac{\;\left(1+2\phi_{d}\right)\;}{\left(1-\phi_{d}\right)} \end{array}$$

As in the Maxwell model no filler-filler particle interactions are considered, the model is only valid for low filler loadings up to a volume fraction of about 0.2 for the filler (ϕ_*d*_) (Pal, 2008).

The volume fraction of the filler in the dispersed phase can be calculated as follows:

$$\begin{array}{l} \phi_{d}=\;\frac{w_{d}/\rho_{d}}{\left(\frac{w_{c}}{\rho_{c}}\right)+\left(\frac{w_{d}}{\rho_{d}}\right)} \end{array}$$

Where, *w*_*d*_ and *w*_*c*_ are the weight percentages, ρ_*d*_ and ρ_*c*_ the densities of the filler (0.451 g^−1^ cm^−3^ for MIL-101Cr) and the polymer (1.17 g^−1^ cm^−3^ for Matrimid), respectively.

The Bruggeman model is based on the effective medium theory and does consider the presence of nearby particles for the permeation properties. Hence, the Bruggeman model is valid for high filler loadings especially.

The permeability for composite membranes can be calculated as follows:

$$\begin{array}{l} {\left(\frac{P_{c}}{P_{eff}}\right)}^{\frac{1}{3}}=\left(1-\phi_{d}\right)\cdot \left(\frac{P_{d}}{P_{c}}-1\right) \end{array}$$

For fillers with distinctly higher permeabilities compared to the continuous phase (*P*_*d*_ >> *P*_*c*_) the equation simplifies to:

$$\begin{array}{l} P_{eff}=\;\frac{P_{c}}{{\left(1-\phi_{d}\right)}^{3}} \end{array}$$

### Dynamic Light Scattering

We performed DLS measurements at different scattering angles θ and thus at different magnitudes of the scattering vector q:

$$\begin{array}{l} |\vec{q}|=\frac{4\pi n}{\lambda }\;\sin\frac{\theta }{2} \end{array}$$

Here λ corresponds to the wavelength of the incident light and n to the refractive index of the dispersing medium. Analysis of the q-dependence of the relaxation rate Γ allows for a diffusion analysis.

For purely translational diffusion, Γ scales with the square of q:

$$\begin{array}{l} {\text{D}}_{\text{t}}=\frac{\Gamma }{q^{2}} \end{array}$$

The diffusion coefficient D_t_ can then be used to calculate the hydrodynamic radius R_h_ of the particles in dilute dispersion using the Stokes–Einstein equation (Cao, 2003):

$$\begin{array}{l} R_{h}=\frac{kT}{6\pi \eta D_{t}} \end{array}$$

Here k is the Boltzmann constant, T the absolute temperature and η the solvent viscosity.

### Synthesis of NanoMIL 101Cr in Direct Emulsion

The chosen parameters led to positive results using our microwave reactor and the tubes provided for this purpose. It should be mentioned, that other research groups with different microwave reactors/tubes might have used different parameters.

Direct emulsion contains DI water, *n*-heptane, surfactant, HNO_3_, and the starting material for MIL-101Cr. First, a solution of H_2_BDC (0.33 g, 2 mmol), Cr(NO_3_)_3_·9H_2_O (0.80 g, 2 mmol), 5 mL water, and 1.0 equivalent of HNO_3_ (with respect to chromium nitrate) were mixed together. This solution was combined with a solution of sodium oleate (0.14 g, 0.34 mmol), 5 mL of water, and ultrasonicated with *n-*heptane (5 mL) for 1 min. The pH value was 4. After the ultrasonication the solution was transferred into microwave ractor tubes with a volume of 55 mL (2.1 cm diameter, height 19 cm). The direct emulsion was heated to 180°C within 30 min and kept at this temperature for 10 min (another batch was kept at 180°C for 40 min). Other direct emulisons were prepared as discribed above but heated to 210°C for 30 min and kept at 210°C for 10 min (another batch for 40 min). After cooling to RT within 15 min the green MIL-101Cr powder was collected by centrifugation and purified as follows: Washing with water (1.00 g MOF to 1.50 L water) by stirring the dispersion for 24 h in order to remove the sodium oleate. Then, the product was washed with hot DMF and hot ethanol two times (2 × 50 mL, 24 h) to remove remaining terephthalic acid. The product was activated under vacuum (50 mbar) at 150°C for 24 h. The reactions were carried out several times to ensure reproducibility. Syntheses without the presence of HNO_3_, syntheses by replacing HNO_3_ with NaOH (to get pH 7), syntheses without *n-*heptane, and direct emulsion in combination with CTAB and Triton X-45 yielded no MIL-101Cr product.

### Attempted Synthesis of NanoMIL 101Cr in Reverse Emulsion

In a one-micellar system, where all reactants are in one micelle, the reaction starts by increasing the temperature or triggering the reaction through other parameters (Anjali and Basavaraj, 2018). Because of the fact that at high temperature micelles coalesce anyway, there is no point in separating the MOF precursors in different micelles at the beginning. Therefore, we suspended all MOF precursors in the same aqueous phase before forming the emulsion (Figures S5, S6).

The reverse emulsion experiments were carried out in a water-in-oil emulsion of chromium(III) nitrate non-ahydrate, terephthalic acid, and nitric acid in a PTFE- (Teflon-) lined vessel at 180°C for 70 min reaction time under microwave heating.

A solution of H_2_BDC (0.33 g, 2 mmol), Cr(NO_3_)_3_·9H_2_O (0.80 g, 2 mmol), 4 mL DI water, and 1.0 equivalent HNO_3_ (with respect to chromium nitrate) were mixed together. This solution was combined with a solution of one of the modulators, sodium oleate (1.00 g, 3.28 mmol), CTAB (1.00 g, 2.73 mmol), or Triton X-45 (1.00 g, 2.47 mmol) in 4 mL of DI water and ultrasonicated with *n*-heptane (40 mL) for 1 min. The pH value was 4. After the ultrasonication the solution was dived into three parts and all transferred into microwave ractor tubes with a volume of 55 mL (2.1 cm diameter, height 19 cm). The reaction was initiated by an increase in temperature up to 180°C for 30 min (heating ramp) in closed 55 mL Teflon vessels by microwave heating at 600 W. After finishing the heating ramp the reaction was hold at 180°C for 40 min (dwelling time). Further steps follow the same route mentioned above. It should be mentioned that with lower amounts of sodium oleate the stability of the direct emulsion gets lost and with a higher amount of sodium oleate or with an increase of temperature higher than 210°C only amorphous material was obtained. To verify the relevance of an acidic modulator, we tried to synthesize nanoMIL-101Cr according to the direct emulsion method using NaOH instead of HNO_3_. We also tried to synthesize nanoMIL-101Cr according to the direct emulsion method without HNO_3_. Also we tried to carry out the synthesis without heptane and just in water with additives. After microwave heating there was no MIL-101Cr material at all. Different variations of direct/reverse emulsions are listed in Supplementary Material.

### Synthesis of MicroMIL 101Cr With TMAOH (Conventional Synthesis)

The hydrothermal synthesis of MIL-101Cr was adapted from the literature (Yang et al., 2010). H_2_BDC (0.332 g, 2 mmol) was added to an alkali solution of 10 mL deionized water (DI water) and tetramethylammonium hydroxide (5 mL, 0.05 mol/L) and stirred at RT for 5 min. Cr(NO_3_)_3_·9H_2_O (0.8 g, 2 mmol) was added to the mixture and stirred for 5 min. The resulting mixture was transferred into a 20 ml Teflon-lined autoclave for 24 h at a heating temperature of 180°C. The green powder was collected by repeated centrifugation and thorough washing with 50 mL DMF at 150°C, 50 mL ethanol at 100°C, and 50 mL DI water at 100°C. The product was activated under vacuum at 150°C for 24 h.

### Preparation of Matrimid Membranes

For the pure Matrimid membrane a certain amount of polymer and DCM were mixed together and stirred for 24 h. The yellowish solution was not further treated before it was cast into 8 cm diameter metal rings, which were placed on a flat glass surface. Next, the rings were covered with funnels with some paper tissue until the membrane was dry (after ~1.5 h).

The funnels were used to prevent contamination by dust particles during the evaporation of the solvent and also to control the evaporation rate. As soon as all solvent was evaporated, the membranes were removed from the metal rings and the glass surface by flushing the rings with deionized water. The membranes were kept in a vacuum oven for another 24 h at 150°C and 50 mbar to remove the residual solvent. After the membranes were naturally cooled to ambient temperature they have been tailored to the size of the sample holder (4.5 cm in diameter) and stored in air and RT for characterization and permeation tests. Details for the specific amounts of polymer and solvent are listed in **Table 2**.

We note that the permeability of pure Matrimid membranes is heavily dependent on the thermal pretreatment of the membrane. Ansaloni et al. (2015) have shown that with higher pretreatment temperature the permeability and also the physical aging of Matrimid is continuously reduced. Also, mixed gas experiments often show reduced permeability compared to single gas experiments due to competitive adsorption and diffusion of the gases. In work by Rodenas et al. (2015), similar experimental conditions were applied (temperature 298 K, transmembrane pressure 3 bar, 50/50 gas mixture CO_2_/CH_4_) and the measured CO_2_ permeability was about 6 Barrer as found by us (**Table 3**). Their slightly lower CO_2_ permeability is in good agreement with their higher pretreatment temperature of 180°C under dynamic vacuum, when compared to our results.

### Preparation of Mixed-Matrix Membranes

For all MMMs nanoMIL-101Cr was used, which was obtained by direct emulsion and sodium oleate (210°C, 70 min). MMMs were fabricated by a dense film casting method using a “priming” technique (Mahajan and Koros, 2002). This technique involves the addition of low amounts of polymer to the MOF-DCM-suspension prior to the incorporation of the particles into the polymer matrix. Priming has shown to support greater affinity between the filler and the polymer, resulting in a symmetric MMM with improved transport properties.

By using the priming technique, a Matrimid-DCM-solution was prepared for each MOF/Matrimid membrane sample (8, 16, and 24 wt.%) and stirred for 24 h. This solution was then combined with an already prepared nanoMIL-101Cr-DCM-suspension in two steps. For the MOF-DCM-suspension a certain amount of MIL-101 was suspended in DCM and stirred for 24 h. Then, the MOF-suspension was sonicated for 15 min with high power ultrasound before stirring it for another 45 min. This procedure was repeated three times. Next, the green suspension was combined with a specific fraction of the previously prepared Matrimid-DCM-solution and stirred again for 24 h (corresponding to the first step of priming). After that, the blend was sonicated for 15 min and stirred for 45 min (here also a repetition of another two times was conducted). Before casting, the leftover Matrimid-DCM-solution was combined with the MOF-DCM-Matrimid-suspension and kept under stirring for 1 h. This final suspension was casted into metal rings. After the solvent was evaporated the membranes were detached, tailored, dried overnight and stored as described above. Details for the specific amounts of MOF, solvent, and polymer are listed in **Table 2**. The experiments were made on mixed-matrix membranes containing 8, 16, and 24 wt.% of MIL-101. Each type of membrane was prepared and measured three times to provide reliable error estimates.

## Results and Discussion

### MIL-101Cr Syntheses

MIL-101Cr (MIL = Matériaux de l'Institute Lavoisier) has become one of the most important MOFs (Férey et al., 2005; Zhao et al., 2015).

In order to decrease reaction time and particle size we tested microwave heating in combination with emulsion techniques. Nanosized particles of MIL-101Cr can be synthesized by microwave heating alone (Khan et al., 2011), yet in our hands the use of microwave heating alone did not lead to reproducible results. Although there are syntheses published to obtain MIL-101Cr with microwave heating and water alone, we could not synthesize MIL-101Cr adopting the published procedures for our microwave reactor. Therefore, we tried to adjust the syntheses and used the direct and reverse emulsion technique in combination with microwave heating. With both techniques we were able to obtain MIL-101Cr in our microwave reactor. To the best of our knowledge we present here the first report where microwave heating, emulsions, and ultrasonication are used in combination for the synthesis of MIL-101Cr nano/microparticles, leading to good yields (45%) in short reaction times (~70 min) without the use of HF or a steel autoclave. Because no HF was used for the syntheses, the formula of the MIL-101Cr framework should be [Cr_3_(O)(OH)(bdc)_3_(H_2_O)_2_].

**Figure 2:**
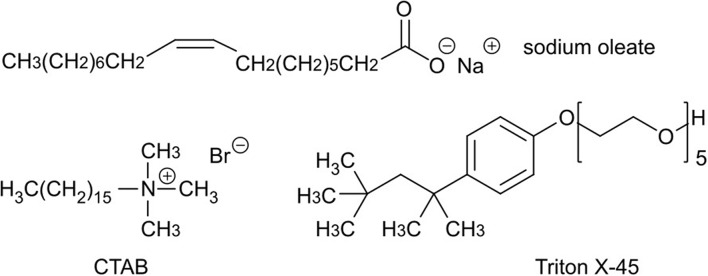
Chemical formulas of the used surfactants sodium oleate, cetyltrimethylammonium bromide (CTAB), and Triton X-45.

For the stabilization of the emulsions we tested three different surfactants, which are generally utilized for MOF emulsion synthesis (Figure 2). Sodium oleate was chosen exemplarily for a monocarboxylic acid and anionic-surfactant, because it should strongly interact with the MOF surface via coordinative bonds and attractive electrostatic forces to open metal sites. This interaction should lead to an adsorption on the MOF surface and inhibits further growth and aggregation (Diring et al., 2010). CTAB was chosen due to its cationic nature and because it is known to slow down the nucleation and growth of MOFs (Jiang et al., 2011b). Triton X-45 was tested as a non-ionic surfactant to achieve additional mesoporosity (Du et al., 2016).

The direct and reverse emulsions were employed under identical temperature and reaction time conditions. All experiments were carried out with 2 mmol each of chromium(III) nitrate non-ahydrate and terephthalic acid in a PTFE- (Teflon-) lined vessel at 180–210°C for 40-70 min (see Supplementary Material). Using direct emulsion, we mixed the MOF precursors and the surfactant together in a heptane-in-water emulsion; for reverse emulsion in a water-in-heptane emulsion. The emulsions were ultrasonicated before heating, otherwise heptane and water would quickly separate again as bulk phases. Ultrasonication achieved sufficient dispersion of the respective droplets in the continuous phase yielding long-time stable emulsions. Baloch et al. have shown that an increase in ultrasonification time increases the number of *n*-heptane droplets and decreases their average size and the degree of dispersity, hence improves the emulsion quality (Baloch and Hameed, 2005). Once the reaction was finished, the MIL-101Cr powder was purified and activated.

It turned out that only the direct emulsion technique in combination with sodium oleate as surfactant gave reproducible and satisfying yields (45%) together with crystalline products of high apparent BET surface areas around 2,900 m^2^ g^−1^ (Table 1). This result may be based on the phase inversion, whereby the oil-in-water emulsion inverts into a water-in-oil emulsion and provides nanoreactors for MOF particles (Figure 1). From FTIR-ATR the infrared band assignments of sodium oleate can still be seen in nanoMIL-101Cr but only as a minor contribution (Figure S7; Roonasi et al., 2010). Concering the yield we note that in the original synthesis procedure by Férey et al. (2005) the problematic modulator HF was used and a yield of only ~50% was stated after separation of MIL-101Cr from the terephthalic acid. Many small-scale literature syntheses follow this original hydrothermal synthesis procedure with a yield of ~50% (Férey et al., 2005). Hence, yields around 45% are competitive with many literature syntheses of MIL-101Cr. Also, the yields of about 45% are slightly higher than for MIL-101Cr samples synthesized by microwave heating alone (de la Iglesia et al., 2016). The use of microwave heating led to a drastic decrease concerning reaction time (70 min) and temperature (210°C and lower). Attempts to shorten the reaction times to 40 min suffered from a loss in surface area and porosity (García-Márquez et al., 2012; Table 1).

**Table 1:** Reaction conditions and results for the synthesis of nanoMIL-101Cr.

**Conditions,^**a**^ surfactant,^**b**^ temp**.	**Time^**c**^ (min)**		**${\text{S}}_{\text{BET}}^{\text{d}}$** **(m^2^·g^−1^)**	**Size^**e**^ (nm)**	**${\text{V}}_{\text{pore}}^{\text{f}}$** **(cm^3^·g^−1^)**	**Yield^**g**^ (%)**
Direct emulsion						
SO (180°C)	40		1,337	<100	0.55	34
Second batch			1,032		0.50	23
SO (180°C)	70		2,269	<100	1.05	43
Second batch			2,400		1.08	30
SO (210°C)	40		1,744	<100	0.73	37
Second batch			1,555		0.75	25
SO (210°C)^h^	70		2,923	<100	1.32	45
Second batch			2,663		1.19	31
Reverse emulsion						
SO (180°C)	70		561	<100	0.31	36
Second batch			417		0.21	21
CTAB (180°C)	70		862	n.d.	0.42	20
Second batch			706		0.35	33
TX-45 (180°C)	70		401	n.d.	0.21	22
Conventional synthesis (see Supplementary Material for details)						
MicroMIL-101Cr^h^	24 h		2,741	350 ± 70	1.25	64

a *All synthesis and other details are given in the Supporting Information. Stated reactions have been carried out in duplicate to ensure reproducibility*.

b *SO, sodium oleate; CTAB, cetyltrimethylammonium bromide; TX-45, Triton X-45*.

c *Time for heating ramp plus dwelling time*.

d *Apparent BET surface area calculated in the pressure range 0.05 < p/p_0_ < 0.2 from N_2_ sorption isotherms at 77 K with an estimated standard deviation of ±50 m^2^ g^-1^*.

e *Particle size based on SEM pictures, for microMIL-101Cr statistic based on 50 particles*.

f *Total pore volume calculated from N_2_ sorption isotherms at 77 K (p/p_0_ = 0.4) for pores ≤ 3.2 nm*.

g *Yield is based on Cr*.

h *Used in mixed-matrix membrane fabrication*.

*n.d. Not determined*.

For comparison, nanoMIL-101Cr was synthesized via reverse emulsion and microMIL-101Cr via the conventional hydrothermal route as described in Supplementary Material. Particles of nanoMIL-101Cr via reverse emulsion were small, but displayed poor porosity characteristics (Table 1).

**Figure 3:**
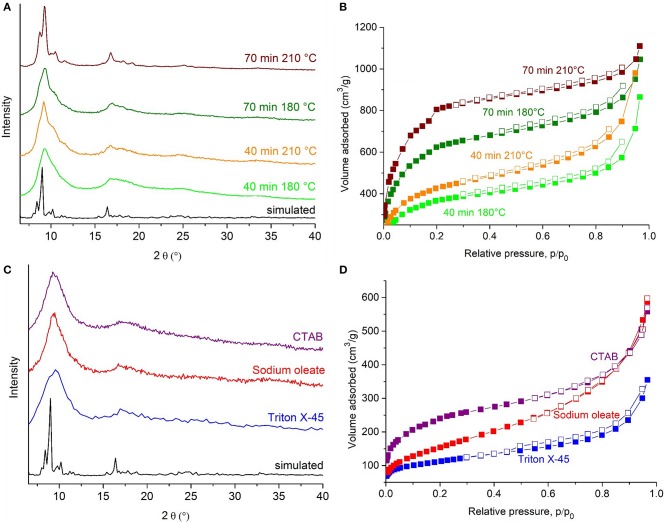
PXRD patterns and N_2_ sorption isotherms of nanoMIL-101Cr synthesized in direct emulsion **(A,B)** for 40 min at 180°C (light green), 40 min at 210°C (orange), 70 min at 180°C (dark green), 70 min at 210°C (brown). PXRD patterns and N_2_ sorption isotherms of nanoMIL-101Cr synthesized in reverse emulsions **(C,D)** by using sodium oleate (red), CTAB (purple), Triton X 45 (blue). Simulated PXRD pattern based on the cif-file of MIL-101Cr, CCDC number: 605510, (black) (Férey et al., 2005). For the N_2_ sorption isotherms the samples were activated at 150°C under vacuum for 12 h. Filled symbols depict adsorption, empty symbols desorption.

Powder X-ray diffraction (PXRD) confirmed the identity, crystallinity and phase purity of nanoMIL-101Cr synthesized using direct emulsions (Figure 3A). It is observed that a higher temperature (210°C) and longer reaction time (70 min) improved crystallinity and porosity (Table 1). As Burrows et al. already have described, the broad Bragg reflections of the XRD patterns of the samples are attributed to the small particle size effects, and indeed the lines get broader as the nano/microparticle size decreases (Jiang et al., 2011a). The porosity characteristics were determined from N_2_ adsorption-desorption isotherms of the purified and activated nanoMIL-101Cr (Figure 3B). The apparent BET surface area could be increased up to 2,900 m^2^ g^−1^ by a 70 min synthesis at 210°C.

The N_2_ isotherms of the higher surface area samples after 70 min reaction time are of the Type I(b) up to p/p_0_ ≈ 0.4 with the characteristic step between 0.1 < p/p_0_ < 0.2 due to the presence of the two kinds of microporous windows/mesoporous cages as in pure MIL-101Cr (Férey et al., 2005). For p/p_0_ > 0.4 the increasing uptake which appears to increase without limit when p/p_0_ = 1 is the result of unrestricted monolayer-multilayer adsorption due to the macroporous voids in the interparticle space so that the isotherm becomes of Type II (Thommes et al., 2015).

Hence, we determined the pore volume at p/p_0_ = 0.4 and not as suggested in the latest IUPAC report for gas sorption at p/p_0_ = 0.95, in order not to overestimate the pore volume (Thommes et al., 2015). Micrometer sized MIL-101Cr from the literature exhibits the typical Type I(b) isotherm for MIL-101Cr, reaching its plateau at p/p_0_ = 0.4 (Zhao et al., 2015). Therefore, it is ensured that pore filling is already completed at p/p_0_ = 0.4. Possibly due to retained surfactant, as mentioned above, the pore volume is smaller than expected. The NL-DFT calculations yielded pore widths largely below ~25 Å (2.5 nm) (Figure S8), which is smaller than the expected 2.9 and 3.4 nm (Figure S4B) but match with literature reports (Horcajada et al., 2007) and with our results on microMIL-101Cr from conventional hydrothermal syntheses (Figure S10; Huang et al., 2012). With shorter reaction times of 40 min not only does the porosity decrease, but the pore size distributions extent also into the mesopore region (above 20 Å, 2 nm; Figures S8A,C). Thus, products from short reaction times seem to have a hierarchically porosity with micropores below 20 Å width and pores sizes between 20 and 100 Å (Figure S11).

From reverse emulsion no satisfactory BET surface area, crystallinity and yield could be reached compared to direct emulsion and conventional syntheses. These results may be based on the phase inversion, whereby the water-in-oil emulsion inverts into an oil-in-water emulsion and as a consequence nanoreactors for MOF particles are no longer available (Figure S6). From PXRD it is evident that the crystallinities of the samples synthesized in reverse emulsion were all very poor (Figure 3C). Still the PXRD patterns with strongly broadened reflections are similar to those for nanosized MIL-101Cr particles reported by other researchers, when using microwave heating or surfactants (Table S1). Broad Bragg diffraction peaks may indicate either a low crystallinity or small (nano)particles (de la Iglesia et al., 2016).

Nitrogen sorption experiments of nanoMIL-101Cr from reverse emulsions yield low BET surface areas of <900 m^2^ g^−1^ and total pore volumes of <0.2 cm^3^ g^−1^ (Table 1, Figure 3D). The low porosity values are in accordance with the lower crystallinity deduced from the PXRD patterns and are in about the same range as those for nanosized MIL-101Cr particles reported by other researchers, when using microwave heating or surfactants (see summary in Table S1). The nitrogen adsorption isotherms are a combination of Type IV at low p/p_0_ (for mesoporous solids) and Type II at high p/p_0_ (Thommes et al., 2015), which is characteristic for macrporous solids with interparticular porosity (Figure S9). When the reverse emulsion is exposed to high temperature (>100°C), heptane vaporizes and expands rapidly, thereby the surface area of the oil within the water increases and an enhanced contact of surfactant molecules with metal salt and organic linker components occurs. This can lead to further competition concerning the coordination equilibrium on the crystal surface, especially for the monocarboxylic acid sodium oleate. All reverse emulsion samples show pore size distributions about 1 nm (10 Å) to over 10 nm (100 Å) with a large pore volume fraction, especially for nanoMIL-101Cr from sodium oleate in the lower mesopore region (between 2 and 10 nm) (**Figure 9A**). Yields of MIL-101Cr from reverse emulsion were below 36% (Table 1).

**Figure 4:**
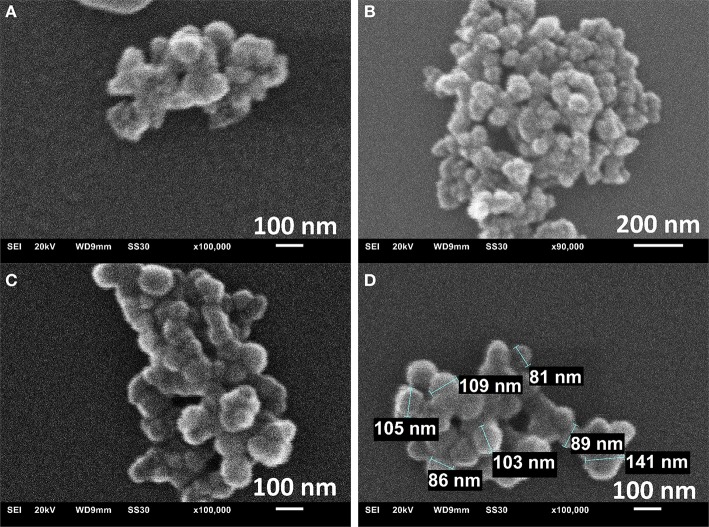
SEM images of the sample prepared in direct emulsion for 40 min at 180°C **(A)**, 70 min at 180°C **(B)** and 70 min at 210°C **(C,D)**.

The morphology and particle size were assessed by scanning electron microscopic (SEM), transmission electron microscopy (TEM) and dynamic light scattering (DLS). SEM images of MIL-101Cr from direct emulsion with sodium oleate show the formation of nano/microparticles with rather uniform diameters around 100 nm and spherical morphology (Figure 4).

**Figure 5:**
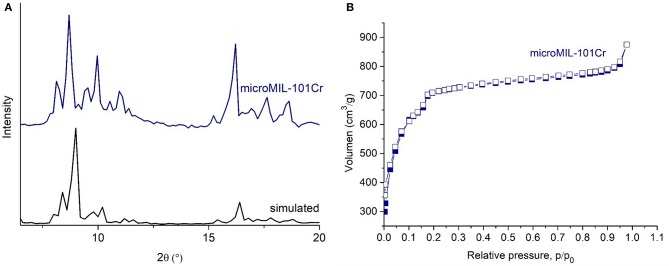
PXRD patterns (navy blue for microMIL-101Cr **(A)**, black for simulated PXRD pattern from the cif-file of MIL-101Cr, CCDC number: 605510) (Férey et al., 2005). N_2_ sorption isotherms of microMIL 101Cr **(B)**. Filled symbols depict adsorption, empty symbols desorption.

TEM images agree with sizes obtained from SEM (Figures S12, S13). The TEM-histograms of nanoMIL-101Cr particles (Figures S12E, S13E) show that the average particle size and its standard deviation are 73 ± 15 and 86 ± 15 nm diameter for direct emulsion at 210°C for 40 and 70 min reaction time, respectively. TEM images are similar to other TEM images already published by other research groups (Wuttke et al., 2015). For MIL-101 powder gained by reverse emulsion the SEM results, TEM results, and a histogram are shown in Figures S14, S15, respectively. For microMIL-101Cr the PXRD, N_2_, SEM results, and a histogram are shown in Figures 5A,B and Figure S16, respectively.

We also used dynamic light scattering (DLS) as a non-destructive ensemble measurement technique to determine the hydrodynamic radius of particles from dilute dispersion. The SEM and TEM images showed aggregates of the small primary particles that may have formed during sample preparation. Since the intensity of scattered light in DLS scales with the diameter at the 6th power, DLS is extremely sensitive to the presence of aggregates or impurities like dust. Indeed, the recorded intensity-time autocorrelation functions and the corresponding distribution functions of the hydrodynamic radii G(R_h_) obtained from CONTIN analysis shown in Figures S17–S23 reveal the presence of small fractions of larger aggregates in all samples. These aggregates appear with average sizes on the order of a few microns. However, we want to note that these larger contributions have low intensities and are clearly not the dominating species in the samples. The diffusion and size of the dominating species [strongest peak in G(R_h_)] in DLS were analyzed using angle dependent measurements. For each sample we determined the mean relaxation rate and observed a linear dependence on the square of the magnitude of the scattering vector q indicating that we only probe translational diffusion. This allowed us to precisely determine the hydrodynamic radii. For the different nanoMIL-101Cr samples from direct emulsion with sodium oleate DLS gave rather uniform hydrodynamic radii between 137 and 155 nm (274–310 nm in diameter). It is also evident that by increasing the reaction temperature from 180 to 210°C or decreasing the reaction time from 70 min to 40 min no significant change of particle size was observed. The determined hydrodynamic radii match nicely to the sizes determined from electron microscopy although slightly smaller values were determined in the latter. This deviation is indeed expected as DLS probes the diffusion of the particles including their solvating shell (Khan et al., 2010).

### MIL-101Cr@Matrimid Mixed-Matrix Membrane

The mixed-gas permeation performance of neat Matrimid membranes and MIL-101Cr MMMs were investigated with a binary gas mixture of CO_2_ and CH_4_. Mixed-gas studies instead of single-gas studies are preferred, since the ideal selectivities from single-gas studies can differ from those of mixed-gases due to gas interaction and plasticization effects (Dechnik et al., 2017b). Plasticization describes the effect that the permeability of both gases increases and the selectivity decreases. This is due to an increase in the segmental motion of polymer chains at high feed pressures caused by the presence of one or more gas sorbates (Wind et al., 2002), CO_2_ in particular causes such plasticization, less so CH_4_ or N_2_. Previous investigations have shown that glassy polymer membranes, including Matrimid, undergo plasticization phenomena only with pressures well above 10 bar (Ismail and Lorna, 2002). No plastization of Matrimid has been reported at a feed pressure of 4 bar where we operated. Comparison of single-gas permeation with CO_2_ and CH_4_ to mixed-gas permeation with a 50:50 v:v CO_2_/CH_4_ mixture at otherwise nearly unchanged conditions for a [Co_4_(μ_4_-O)(Me_2_pzba)_3_]-MOF@Matrimid-MMM revealed only a slightly reduced permeability for the latter mixed-gas measurement. At the used transmembrane pressure of 3 bar plasticization could be ruled out (Dechnik et al., 2017c).

Neat Matrimid membranes and MIL-101Cr@Matrimid MMMs were fabricated with different loadings of the MOF (8, 16, and 20 or 24 wt.%). For comparison, nanoMIL-101Cr from direct emulsion with sodium oleate and microMIL-101Cr from conventional hydrothermal conditions were used to prepare nanoMIL-101Cr@Matrimid MMMs and microMIL-101Cr@Matrimid MMMs, respectively. For the MMM fabrication chosen amounts of MOF and Matrimid (Table 2) were first mixed with dichloromethane, DCM, and stirred, before the suspension was casted into metal rings. After covering the rings with funnels and evaporation of the solvent (Figure S24), the membranes were detached, tailored, dried and stored. The detailed procedure is described in Supplementary Material.

**Table 2:** Weight and mass content of Matrimid and MOF in mixed-matrix membranes.

**MOF (wt.%)**	**Matrimid (g)**	**DCM (Matrimid) (ml)**	**MOF [g]**	**DCM (MOF) (ml)**	**Matrimid-DCM^**a**^ for priming (ml)**
0	0.80	7.0	–	–	–
8	0.80	7.0	0.07	9.0	0.67
16	0.80	7.0	0.15	9.0	1.34
24	0.80	7.0	0.25	9.0	2.01

a *Taken from the prepared Matrimid-DCM solution*.

A major difference was already noticed for the preparation of the highly loaded MMMs where it was not possible to produce microMIL-101Cr-MMMs with 24 wt.% MOF because of their extreme brittleness (Figures S24B,C). Hence, we could only produce and measure microMIL-101Cr@Matrimid MMMs with 8, 16, and 20 wt.% MOF loading.

The XRD diffractogram of pure Matrimid shows a broad reflection around 2θ = 20° with low intensity based on the semi-crystalline structure (Amooghin et al., 2015). For MMMs, all the intense reflections of MIL-101Cr and the broad reflection of Matrimid can be observed (Figure S25A). The MIL-101 reflections confirm that the MOF material were not affected by the preparation of the membranes. For details regarding the infrared analysis of the MMMs see Figure S26.

**Figure 6:**
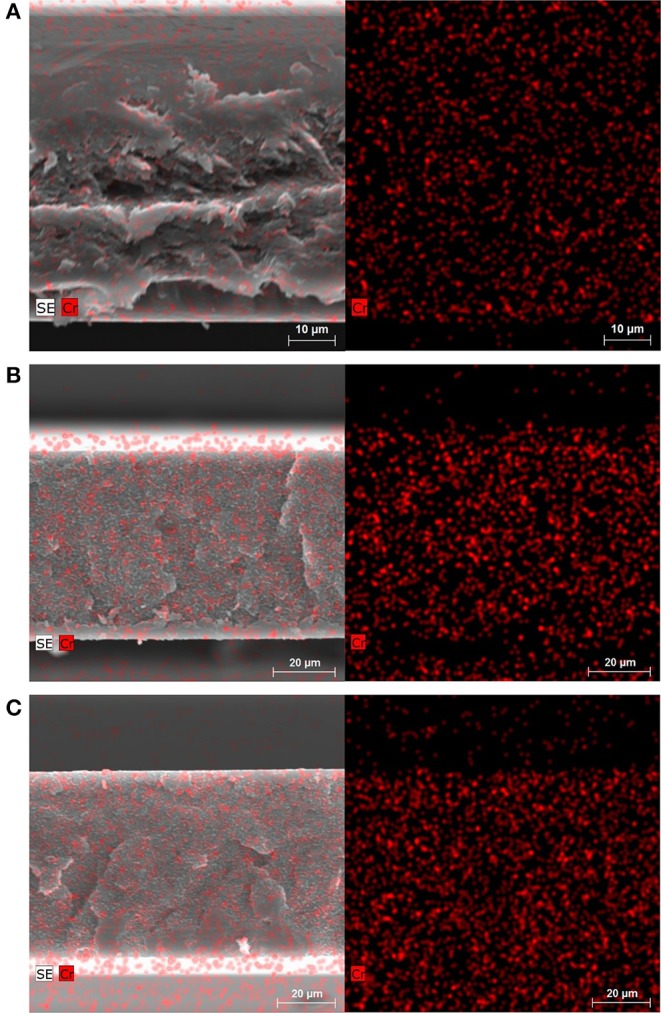
SEM/EDX images of the cross-section of nanoMIL-101Cr@Matrimid membrane with 8 wt.% MOF **(A)**, 16 wt.% MOF **(B)**, and 24 wt.% MOF **(C)**. Left: SEM/EDX images superimposed. Right: EDX element mapping (Cr = red). The bottom of the cross-sections in the image corresponds to the bottom of the membrane when casted. The cross-section of a pure Matrimid membrane is shown in Figure S25B. Dark and light areas around cross-section of membranes depend on mounting on SEM sample holder (see Figure S2).

SEM images in combination with energy-dispersive X-ray spectroscopy (EDX) of cross-sections of the membranes confirm the homogeneous distribution of nanoMIL-101Cr particles (Figure 6). The uniform distribution is due to the organic-inorganic structure of the MOF filler particles, which disperse well within the continuous polymer phase. SEM images of cross-sections and EDX of the membranes fabricated with conventional synthesized microMIL-101Cr are shown in Figure S26. Even larger microMIL-101Cr in Matrimid MMMs lead also to a homogeneous distribution in the polymer matrix.

### Mixed-Gas Permeation Performance

**Figure 7:**
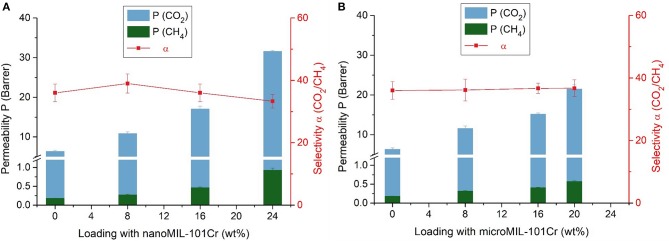
Separation properties of nanoMIL-101Cr@Matrimid **(A)** and microMIL-101Cr@Matrimid **(B)** MMMs with different MOF loadings for a 50:50 v:v gas mixture of CO_2_ and CH_4_ at 25°C and a feed pressure of 4 bar. Error bars of the permeability correspond to measurement uncertainties of the three investigated membranes; error bars of the selectivity were calculated via error propagation, that is, by summation of the relative errors of the permeabilities.

The mixed-gas permeation performance of neat Matrimid membranes and mixed-matrix membranes were investigated with a binary gas mixture of CO_2_ and CH_4_. Before the measurement, the membranes were again heated for 1 h in a vacuum oven at 150°C. The results for the permeability and selectivity for the MIL-101Cr@Matrimid membranes are shown in Table 3 and Figure 7.

**Table 3:** Summary of gas permeation properties of nanoMIL-101Cr@Matrimid and MIL-101Cr@Matrimid membranes for a 50:50 v:v gas mixture of CO_2_ and CH_4_.^a^

	**Membrane**	**MIL-101 loading**	***P* (CO_**2**_) (Barrer)^**b**^**	***P* (CH_**4**_) (Barrer)^**b**^**	**S (CO_**2**_/CH_**4**_)^**b**^**
**MOF**	**Thickness^**c**^ (μm)**				
–	59-60	Pure matrimid	6.6 ± 0.3	0.18 ± 0.01	36 ± 3
NanoMIL-101Cr	68-70	8 wt.%	10.8 ± 0.4	0.28 ± 0.01	39 ± 3
	69-79	16 wt.%	17.0 ± 0.7	0.47 ± 0.02	36 ± 3
	78-88	24 wt.%	31.6 ± 0.2	0.93 ± 0.06	33 ± 2
MicroMIL-101Cr	67-69	8 wt.%	11.6 ± 0.6	0.31 ± 0.01	36 ± 4
	75-75	16 wt.%	15.2 ± 0.3	0.41 ± 0.01	37 ± 2
	70-81	20 wt.%	21.5 ± 0.1	0.58 ± 0.01	37 ± 3

a *At 25°C and a feed pressure of 4 bar. Each type of membrane was prepared and measured three times to provide reliable error estimates*.

b Error margins for CO_2_ and CH_4_ permeabilities were deduced from measurement uncertainties of the three investigated membranes. Error margins for the CO_2_/CH_4_ selectivity were calculated by error propagation, that is, by summation of the relative errors of the permeabilities.

c *Each membrane was measured at 10 different places using a thickness measuring device*.

Nano- and microMIL-101Cr@Matrimid MMMs show a similar rise in CO_2_ and CH_4_ permeability with increasing MOF loading. Furthermore, within the measurement error margin the selectivity remains unaltered for all measured MMMs. By comparing our results for CO_2_ and CH_4_ permeability with already published results (Table S2) it can be noticed, that with our nanoMIL-101Cr mixed-matrix membranes we have achieved very high CO_2_ and CH_4_ permeability results with an almost steady selectivity. By taking into account the results for selectivity of the already published studies it is obvious that their selectivity results are sometimes high sometimes low without any correlation concerning the MOF loading. Therefore, the achievement of almost steady selectivity without great changes is worth to be mentioned.

However, we note again that it was not possible to produce MMMs with 24 wt.% microMIL-101Cr. The CO_2_ permeability increased from about 6 Barrer for pure Matrimid to over 31 Barrer for the 24 wt.% nanoMIL-101Cr membrane, but to only 21 Barrer for the 20 wt.% microMIL-101Cr membrane. Thus, at 24 wt.% loading of nanoMIL-101Cr, the permeability of the membrane for both gases increased almost five times with respect to the pure Matrimid membrane. However, at 20 wt.% loading of microMIL-101Cr, the permeability for both gases increased only three times with respect to the pure Matrimid membrane. The permeability increase can be traced to the added free fractional volume, which is introduced by the incorporation of the porous MIL-101Cr filler particles. Furthermore, one can observe only a negligible reduction in the CO_2_/CH_4_ selectivity of around 36 for both MOF variations.

**Figure 8:**
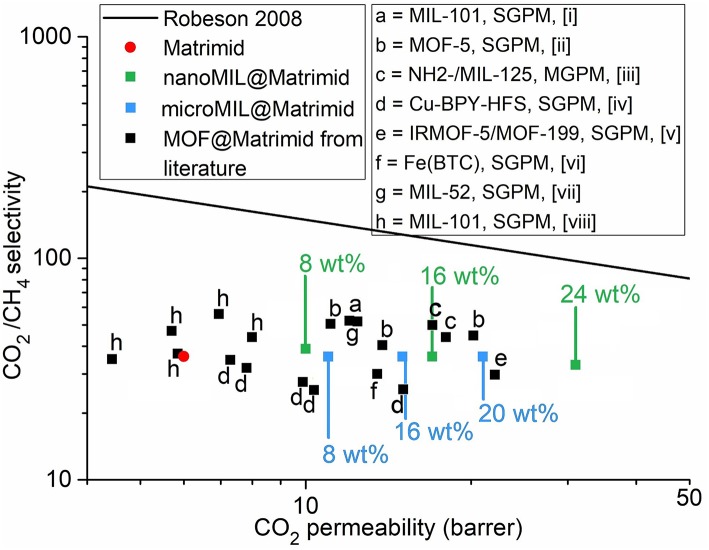
CO_2_/CH_4_ separation performance of nanoMIL-101Cr@Matrimid (green), microMIL-101Cr@Matrimid (light blue), and neat Matrimid (red) compared with published results for mixed-matrix membranes based on MOF@Matrimid (black). The upper bound for polymer performances as defined by Robeson in 2008 is given as a black line (Robeson, 2008). SGPM = single-gas permeation measurement. MGPM, mixed-gas permeation measurement. References: a, (Rajati et al., 2018); b, (Perez et al., 2009); c, (Anjum et al., 2016); d, (Zhang et al., 2008); e, (Nik et al., 2012); f, (Shahid and Nijmeijer, 2014); g, (Dorosti et al., 2014); and h, (Naseri et al., 2015).

For comparison, single-gas and mixed-gas permeation measurements for CO_2_/CH_4_ separation of different MOF@Matrimid and MIL-101Cr@polymer MMMs are collected in Table S2. The CO_2_ permeability and CO_2_/CH_4_ selectivity of the membranes fabricated in this study together with MOF@Matrimid MMMs from the literature are summarized in a Robeson chart (Figure 8). From this chart it is obvious that MMMs with MIL-101Cr@Matrimid compare well in performance with already published results. Yet, the nanoMIL-101Cr-MMM with 24 wt.% surpasses all other MOF@Matrimid MMMs in its CO_2_ permeability even though some of these other MOF-MMMs have MOF weight percentages of 30 or even 40 wt.% (Zhang et al., 2008; Perez et al., 2009; Naseri et al., 2015; Rajati et al., 2018).

### Modeling

**Figure 9:**
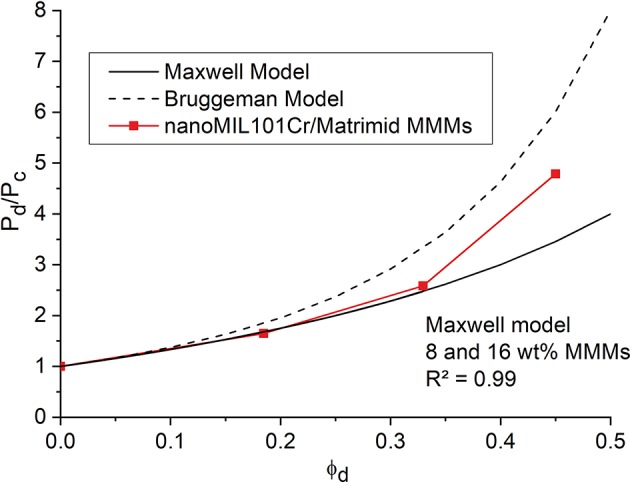
Relative experimental CO_2_ permeabilities (referenced to the permeability P_c_ of the pure polymer membrane) for nanoMIL-101Cr@Matrimid (red curve) with different filler volume fraction ϕ_d_. The black solid line gives the theoretical CO_2_ permeabilities for porous fillers based on the Maxwell Model and the black dashed line gives the theoretical CO_2_ permeabilities for porous fillers based on the Bruggeman Model.

Figure 9 shows the comparison between predicted Maxwell and Bruggeman model (see Supplementary Material for details) and experimental relative CO_2_ permeabilities for nanoMIL-101Cr@Matrimid MMMs. The very high pore volume of 1.32 cm^3^ g^−1^ for nanoMIL-101Cr results in a low crystal density (0.451 g^−1^ cm^−3^) and therefore the values of the weight percentages of 8, 16, and 24 wt.% roughly double when converted into filler volume fraction. Thus, MMMs loaded with 8, 16, and 24 wt.% MOF give a filler volume of 0.18, 0.32, and 0.45, respectively. We note, that for an effective increase in permeability the filler volume fraction (ϕ_*d*_) is of high relevance and is more important than the usually given wt.% loading of the membrane. Further, the filler volume fraction for a highly porous material is largely determined by the free volume (pore volume) of the filler. As found in earlier work (Nuhnen et al., 2018) we can hypothesize again that a high pore volume is decisive for MOFs to yield high permeable MMMs. The relative CO_2_ permeabilities for the MMMs with lower filler loadings follow exactly the predicted values for the Maxwell model. This is in good agreement with previous studies, where composite membranes for filler loadings up to 0.2 and even slightly above mostly follow the Maxwell model (Nuhnen et al., 2018). For higher filler loadings above 0.2 the relative CO_2_ permeabilities starts to deviate from the Maxwell model and approach the calculated values based on the Bruggeman model. Since the Maxwell model does not consider filler-filler particle interaction, it is not suitable for filler loadings significantly above 0.2. In contrary the Bruggeman model is explicitly used for higher filler loadings in MMMs and also considers the effects of filler-filler particle interactions (Shen and Lua, 2013). Hence, the permeation data displays a good agreement for both models in their respective filler loading range. Relative CH_4_ permeabilities show the same trend (Figure S28).

A comparison between the predicted Maxwell and Bruggeman model and the experimental relative CO_2_ and CH_4_ permeabilities for microMIL-101Cr@Matrimid MMMs, presented in Figures S29, S30, yields slightly different results. For lower filler volumes the experimental data follows the Maxwell model and with rising filler volume it only slightly surpasses the values calculated for the Maxwell model, but does not reach the calculation derived by the Bruggeman model. For the studied MMMs it is observed, that the filler volume fraction, where filler-filler particle interactions influence the permeation performance in a significant manner, is somewhere between 0.35 and 0.45. In this range of the filler volume fraction the permeability starts to rise sharply. This shows in the permeation performance of nanoMIL-101Cr@Matrimid MMMs, which is superior to the regular microMIL-101Cr@Matrimid MMMs, as it is possible to reach higher filler volume fractions to achieve this sharp rise in permeability.

Another way to analyze permeation properties, is to take a look at the so called Free Fractional Volume (FFV). The FFV is defined as the sum of the volume weighted FFV of the polymer and the volume weighted FFV of the filler (Equation 1).

$$\begin{array}{l} \left(Total\right)\;FFV=FFV_{polymer}\cdot \;\phi_{c}+\;FFV_{filler}\;\cdot \;\phi_{d} \end{array}$$

Whereby the FFV of the polymer can be deduced by the Bondi method, which is described in detail in the literature and was determined as 0.17 for Matrimid (Huang et al., 2006; Kanehashi et al., 2015). The FFV of the filler can be calculated by multiplying the pore volume of the MOF, here obtained by nitrogen sorption of nanoMIL-101Cr and microMIL101-Cr, with the density of the MOF, to yield a dimensionless entity. Equation (2) shows the correlation of the FFV and the permeability *P*:

$$\begin{array}{l} P=\;A_{p}\cdot \;\exp^{\left(-\frac{B_{p}}{FFV}\right)} \end{array}$$

After linearization:

$$\begin{array}{l} \mathrm{lg}P=\mathrm{lg}A_{p}\;-\frac{B_{p}}{2.303FFV} \end{array}$$

**Figure 10:**
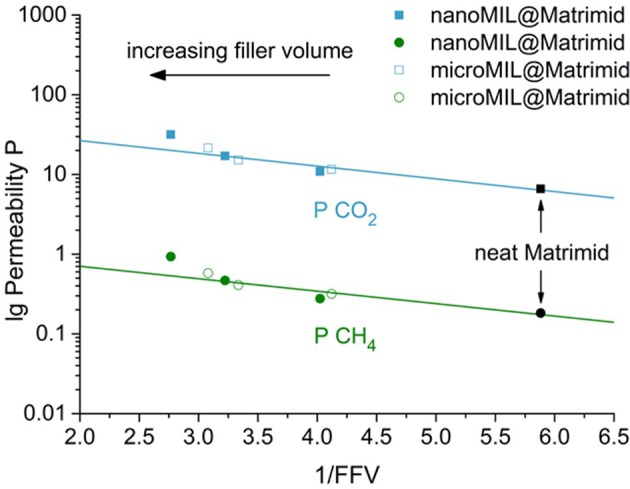
Experimental CO_2_ and CH_4_ permeabilities of nano- and microMIL-101Cr@Matrimid MMMs as a function of the inverse FFV. The neat polymer has 1/FFV = 5.9, the 20 wt.% microMIL-MMMs has 1/FFV = 3.1, the 24 wt.% nanoMIL-MMM has 1/FFV = 2.8. The added straight lines are intended as guides to the eye.

Hence, lg P plotted against the inverse FFV should give a linear correlation with slope – *B*_p_/2.303 and intercept lg *A*_p_. if indeed the FFV determines the permeability. Figure 10 shows the plot of lg P vs. 1/FFV for the nano- and microMIL-101Cr@Matrimid MMMs. As the FFV rises with increasing filler volume, the inverse FFV decreases. For filler volumes between 0 and 16 wt.% MOF the reduction of the inverse FFV and the rise in permeability show a good linear correlation, displayed by the straight line. The nanoMIL-MMM with 24 wt.% MOF however, has a higher than expected permeability. This is in good agreement with the previous observation that for MMMs with 24 wt.% filler loading a sharp increase in permeability takes place which leads to a significant deviation from the used FFV model. As it seems, similar to the Maxwell model, the FFV model is no longer applicable for MMMs with higher filler loadings, where filler-filler interaction starts to occur. Such filler-filler interaction would add an additional FFV (yielding an even smaller 1/FFV value). A similar effect could be observed in the work of Kanehashi et al. (2015) were they plotted lg*P* vs. 1/FFV for several MOFs and carbons materials. For example, a Cu-BTC MMMs with the highest filler loadings of 30 wt.% showed a distinct increase in CO_2_ and CH_4_ permeability compared to the given 1/FFV (Kanehashi et al., 2015).

## Conclusion

In summary, we demonstrated that the size of MIL-101Cr can be adjusted in the nano-micro-range below 100 nm by using microwave heating in combination with a direct emulsion technique, surfactants, and ultrasonication. Direct emulsion with the phase-inversion-temperature method and sodium oleate as surfactant yielded nanoMIL-101Cr particles of <100 nm and high apparent BET-surface areas (2,900 m^2^ g^−1^) in good yields (45%). Furthermore, we observed, that with increasing reaction time and temperature, the particles became more crystalline and showed higher N_2_ uptakes, as well as larger total pore volumes. Thereby we prepared nanoMIL-101Cr for application as a filler in mixed-matrix membranes. The use of nanoMIL-101Cr in Matrimid MMMs allowed for the preparation of 24 wt.% filler MMMs compared to a maximum of 20 wt.% for microMIL-101Cr in order to still have a defect-free membrane. The increased filler amount improved the gas permeability. Permeation modeling studies based on the Maxwell model and the free fractional volumes indicate a significant permeability increase beyond the 20 wt.% filler content. Our approach, to surpass the normally obtainable filler wt.% fraction by a nanosized filler in a mechanically still stable membrane, is a promising result with wider implications.

## Data Availability Statement

All datasets generated for this study are included in the article/Supplementary Material.

## Author Contributions

IG synthesized, fabricated the nano, microsized MOF as well as the mixed-matrix-membranes, performed, discussed the powder X-ray diffraction, nitrogen physisorption, infrared experiments, and wrote the manuscript. AN performed, discussed the mixed-gas permeation, and wrote the manuscript part concerning the mixed-matrix-membranes. AL performed, discussed the DLS experiments, and results given by the 3D LS spectrometer. SN and MKl performed the scanning electron microscopy, SEM, and EDX analysis. AH contributed the idea of emulsion and microwave heating based synthesis of MIL-101 and ran pilot tests. MKa and CJ proofread and refined the manuscript.

### Conflict of Interest

The authors declare that the research was conducted in the absence of any commercial or financial relationships that could be construed as a potential conflict of interest.
