# Supplementary material for: Synthesis of Nano/Microsized MIL-101Cr Through Combination of Microwave Heating and Emulsion Technology for Mixed-Matrix Membranes
Source: Front Chem. 2019 Nov 19;7:777. doi: 10.3389/fchem.2019.00777 (PMC6877507; doi:10.3389/fchem.2019.00777)
Supplement: Supplementary file 1 [file Data_Sheet_1.PDF]

frontiers

Supplementary Material

1 Data Content

2 Instrumentation

3 Additional Data

3.1 Published BET results and particle sizes for MIL 101Cr synthesized via conventional and microwave heating

3.2 Published results for MMMs

4 Structure features of MIL-101Cr

5 Additional information for the syntheses of nano/microsized- and microMIL-101Cr

5.1 Different variations of direct/reverse emulsions

6 Additional analysis for nano/microsized- and microMIL-101Cr

6.1 Fourier transform infrared-attenuated total reflectance spectroscopy (FTIR-ATR)

6.2 Pore-size distribution

6.3 Scanning electron microscopic (SEM) and transmission electron microscopy (TEM)

6.4 Dynamic light scattering (DLS)

7 Mixed-matrix membranes

8 Additional analysis for mixed-matrix membranes

8.1 Powder X-ray diffraction, scanning electron microscopic (SEM), and infrared analysis (IR)

8.2 Maxwell and Bruggeman model

9 References

2 Instrumentation

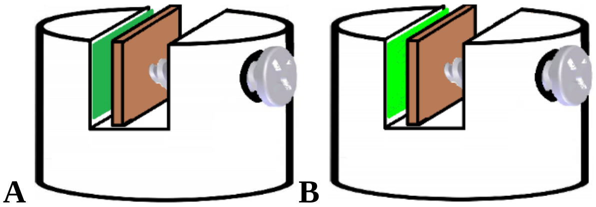

Figure S1 Schematic illustration of a SEM 90-degree sample mount to mount the cross-section of membranes (green/light green squares) directly against the vertical side of the mount. Open slot with the bottom of the cross-section facing the screw and metal sheet (brown square) **(A)** and open slot with the bottom of the cross-section facing away from the screw and metal sheet **(B)**.

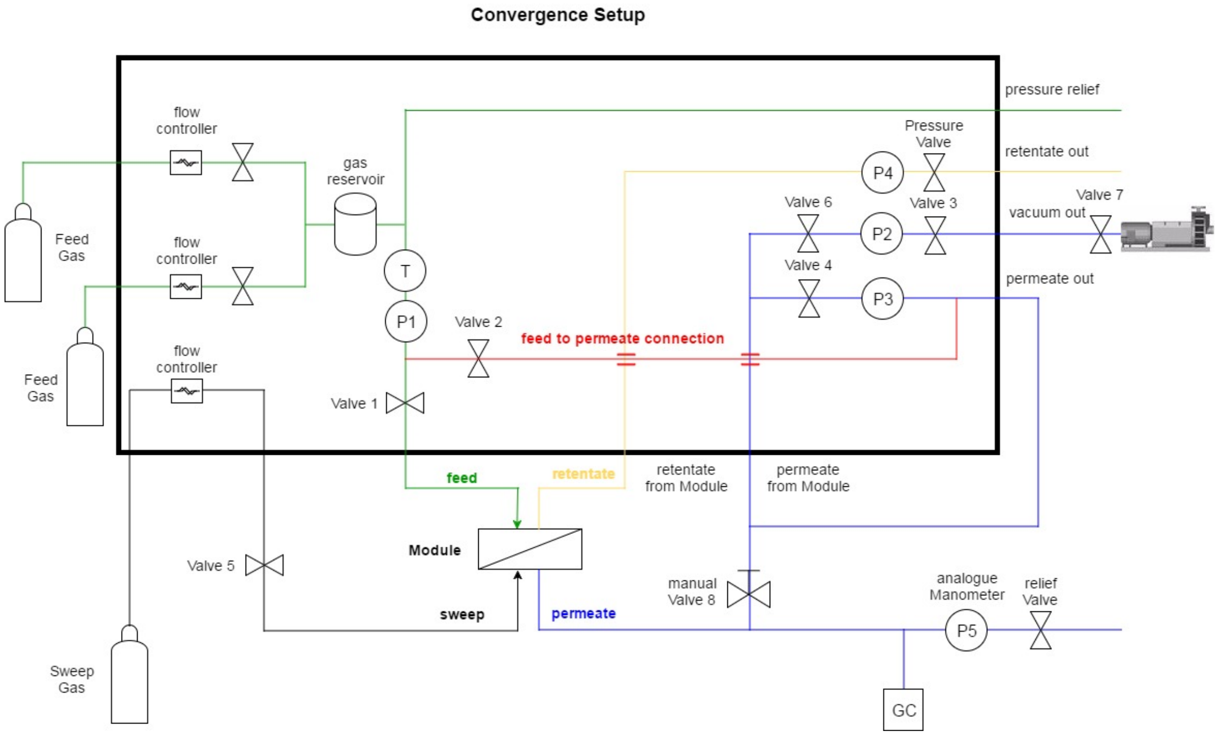

Figure S2 Schematic illustration of the mixed-gas permeation pilot. Black boarded area represents the OSMO Inspector.

3 Additional Data

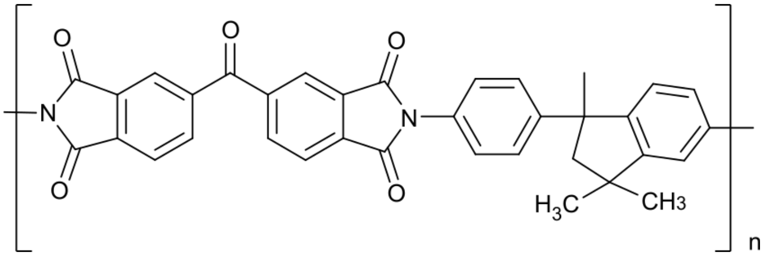

Figure S3 Repeating unit of Matrimid 5218.

3.1 Published BET results and particle sizes for MIL 101Cr synthesized via conventional and microwave heating

The following table (**Table S1**) shows the results for BET and particle sizes as well as additional published results by other working groups with and without the use of modulators. Thereby a possible comparison can be eased.

Table S1 Data for MIL-101Cr synthesized via conventional and microwave heating already published by other groups.

Sample name [a]	Synthesis condition heating/temp./time	Surfactant	Particle size [nm]	BET [$m^2 \cdot g^{-1}$]	Ref.
A1	CE/220 °C/8 h	None	n.d.	2962	i
A2				2439	
A3				2345	
A4				1724	
A5		CTAB	n.d.	846	
A6				609	
B1				619	
B2				717	
C1				465	
C2				1026	
A0	CE/220 °C/12 h	None	n.d.	2341	ii
A1		CTAB	n.d.	1804	
A2				1596	
A3				1560	
B0		None	n.d.	3514	
B2		CTAB	n.d.	1454	
B3				1144	
A	CE/ 210 °C/6 h	None	800	2735	iii
B	CE/ 210 °C/24 h		400	3160	
C	CE/210 °C/ 24 h		200	ND	
D	MW/ 210 °C/15 min		200	3071	
E	MW/ 210 °C/1 h		200	3196	
F	MW/210 °C/1 h		100	ND	
G	CE/ 210 °C/4 h		400	3160	
H	CE/ 210 °C/24 h		100	ND	
J	MW/ 210 °C/15 min		200	3071	
K	MW/ 210 °C/15 min		50	3223	
(a)	CE/180 °C/8 h	None	50 (9)	2944	iv
(b)		Stearic Acid	19 (4)	2691	
(c)		4-Methoxybenzoic acid	25 (6)	2646	
(d)		Benzoic acid	28 (6)	2923	
(e)		4-Nitrobenzoic acid	36 (7)	2692	
(f)		Perfluorobenzoic acid	73 (8)	2893	
MIL-101Cr nanoparticles	MW/180 °C/6 min	None	49±20 nm	3205	v

[a] Sample name according to the reference shown in the right column. n.d. not determined. i = (Huang et al., 2012); ii = (Shen et al., 2015); iii = (Khan et al., 2011); iv = (Jiang et al., 2011); v = (Wuttke et al., 2015).

3.2 Published results for MMMs

Table S2 Gas (CO_2/CH_4) permeation data of mixed-matrix membranes with different MOFs and different polymers from literature.

SGPM MGPM [a]	Polymer	MOF	MOF [wt%]	P(CO_2) [Barrer]	P(CH_4) [Barrer]	S (CO_2/CH_4)	Ref.
SGPM	Matrimid			7.33	0.21	34.90	i
	PVDF			2.11	0.08	26.37	
	Matrimid/PVDF(3%)			9.42	0.22	42.81	
	Matrimid	MIL-101	10	12.01	0.23	52.21	
	Matrimid/PVDF(3%)	MIL-101	10	14.87	0.24	61.95	
SGPM	Matrimid	MOF-5	10	11.1	0.22	50.5	ii
			20	13.8	0.34	40.5	
			30	20.2	0.45	44.7	
MGPM (50:50)	Matrimid	MIL-125	15	18	0.41	44	iii
		NH_2-MIL-125	15	17	0.34	50	
SGPM	Matrimid	Cu-BPY-HFS	0	7.29	0.21	34.7	iv
			10	7.81	0.24	31.9	
			20	9.88	0.36	27.6	
			30	10.36	0.38	25.4	
			40	15.06	0.59	25.6	
SGPM	Matrimid	IRMOF-5	30	20.2	0.45	44.7	v
		MOF-199	30	22.1	0.74	29.8	
SGPM	Matrimid	Fe(BTC)	30	13.5	0.45	30	vi
SGPM	Matrimid	MIL-53	15	12.43	0.24	51.8	vii
SGPM	Matrimid	MIL-101	0	4.44	0.126	35	viii
			10	6.95	0.125	56	
			15	5.70	0.120	47	
			20	5.85	0.155	37	
			30	7.99	0.181	44	

[a] SGPM = single-gas permeation measurement. MGPM = mixed-gas permeation measurement. Permeability data have been obtained out at different feed pressures Results are shown in **Figure 8** in the manuscript. i = (Rajati et. al., 2018); ii = (Perez et. al., 2009); iii = (Anjum et. al., 2016); iv = (Zhang et. al., 2008); v = (Nik et. al., 2012); vi = (Shahid and Nijmeijer 2014); vii = (Dorosti et. al., 2014); viii = (Naseri et. al., 2015);

4 Synthesis and structure features of MIL-101Cr

MIL-101Cr is hydrothermally very stable (Khutia et al., 2013), offers good adsorption of gases (Khutia and Janiak, 2014), water (Wickenheisser et al., 2013), and organic compounds (Rezk et al., 2013), catalytic Lewis- and Brønsted-acid activity (Herbst and Janiak, 2016), and polymer compatibility in mixed-matrix membranes (MMMs) (Tanh Jeazet et al., 2012). The MIL-101Cr crystallites have an octahedral shape, and the framework is built from trinuclear {$Cr_3(\mu_3$-O)(F,OH)$(H_2O)_2$} nodes and 1,4-benzenedicarboxylate (bdc) linkers (**Figure S4A**) to give hexagonal (15-16 Å) and pentagonal windows (12 Å) (**Figure S4B**), inner cages of 29 Å and 34 Å in diameter (**Figure S4C**) (Wickenheisser and Janiak, 2015), and large apparent BET surface area (4100 m^2 g^{-1}) in a zeotypic structure (**Figure S4D**). For the synthesis of MIL-101Cr long reaction times of 8 h, and temperatures in the region of 160 °C to 220 °C with hydrothermal autoclaving are usually needed (Férey et al., 2005). Further, for a typical synthesis of MIL-101Cr hydrofluoric acid is often used as a problematic additive or modifier (Férey et al., 2005). Overpressure (autoclaves) and/or heterogeneous reaction mixtures render reaction control difficult and require expensive equipment, especially when it comes to scale-up, hence, are not desirable for large-scale or commercial syntheses (Jeremias et al., 2016). Therefore, a more efficient synthesis of MIL-101Cr is highly wanted. For example, nitric acid, HNO_3 was also shown to act as mineralizing agent and increased crystallinity and yield (Zhao et al., 2015).

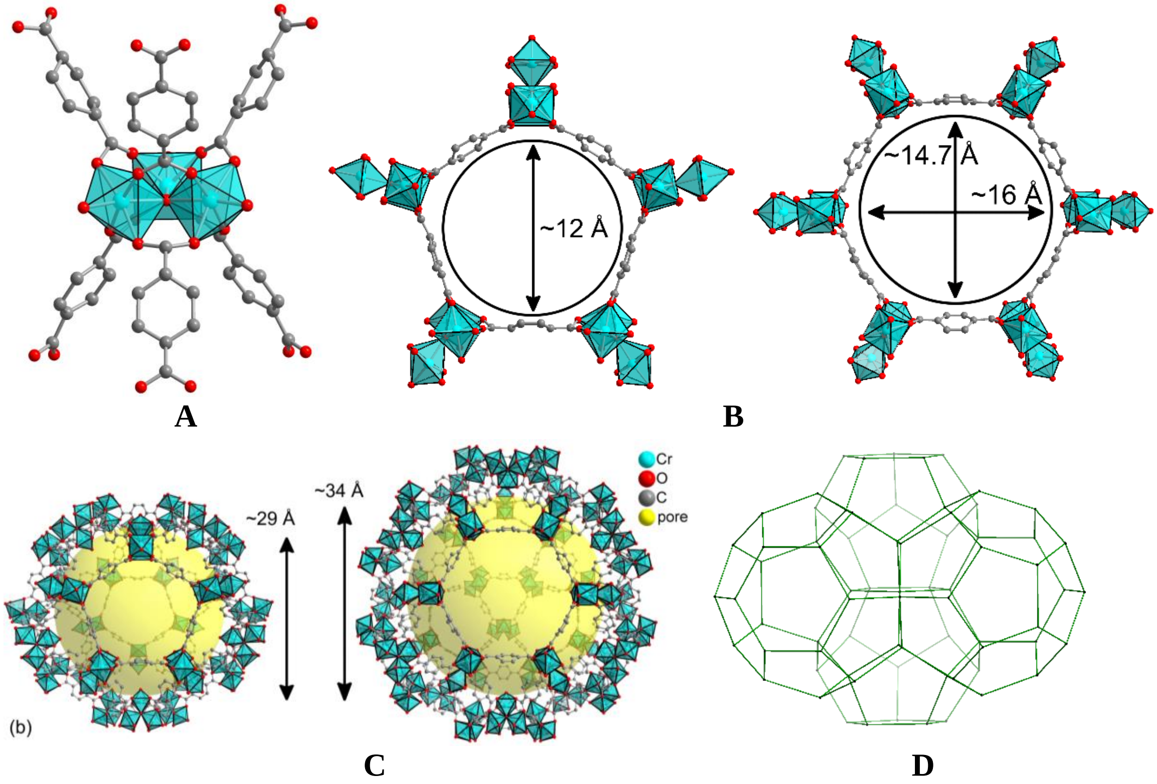

Figure S4 Trinuclear {$Cr_3(\mu_3$-O)$(O_2C$-$)_6$(F,OH)$(H_2O)_2$} building unit surrounded by bdc linkers in a trigonal prismatic fashion **(A)**; pentagonal and hexagonal cage window apertures with dimensions **(B)**; small cage with pentagonal windows and large cage with pentagonal and hexagonal windows **(C)**; zeotypic network **(D)**. Objects in **(A)** to **(D)** are not drawn to scale. Graphics have been created from the deposited cif-file for MIL-101Cr (CSD-Refcode OCUNAK) (Férey et al., 2005).

5 Additional information for the syntheses of nano/microsized- and microMIL-101Cr

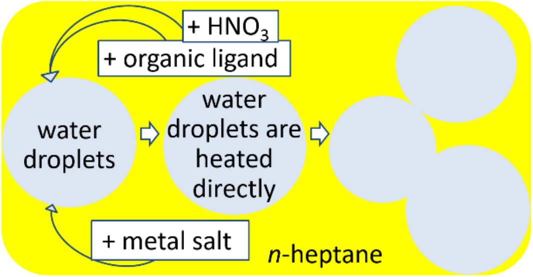

Figure S5 Schematic illustration of water droplets with MOF precursors in heptane of a reverse emulsion. The ater droplets expand and micronize rapidly when heated under microwave irradiation, hence, surface area of water droplets increases, and contact between surfactant and MOF increases (see **Figure S6**).

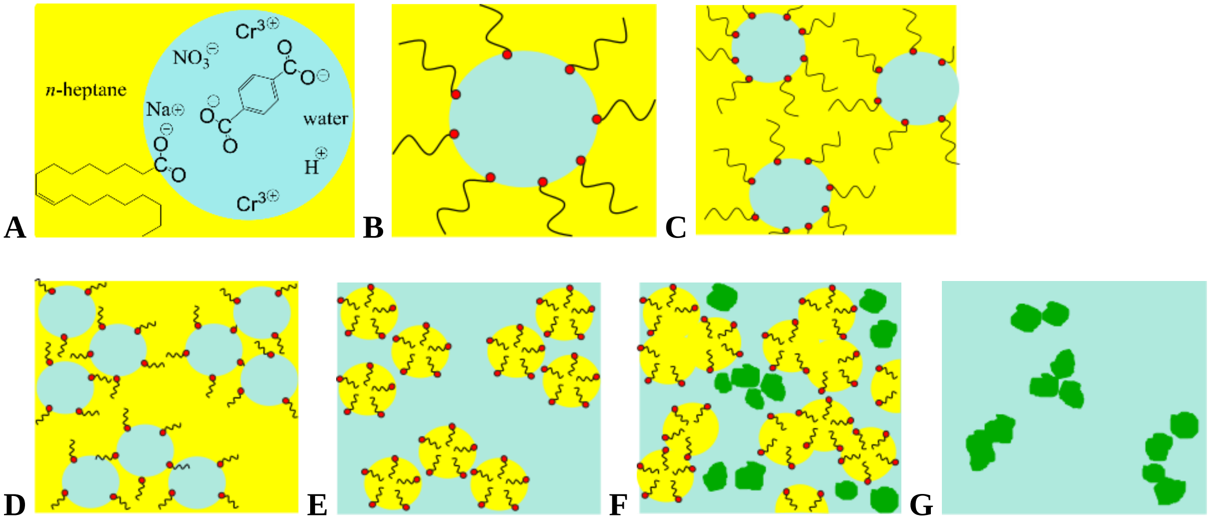

Figure S6 The anticipated reverse emulsion mechanism for the formation of nanoMIL-101Cr (depicted as green domains). BDC^{2-} = benzene-1,4-dicarboxylate, Cr^{3+} = chromium salt, NO_3^- = nitrate ion, and inverse micelles with hydrophobic groups of sodium oleate (hydrophilic groups = red spheres) oriented away from the center (center = water droplets with MOF precursors). Reverse micelle with sodium oleate in n-heptane **(A**,**B)**. At higher temperature the heptane droplets expand rapidly **(C)**. At the PIT (phase inversion temperature) the size of the water droplet reaches a minimum **(D)**, phase inversion occurs **(E)**, coalescence of micelles and formation of nanoMIL-101Cr in the continuous phase **(F)**. Isolated and agglomerated nanoMIL-101Cr particles in water after washing procedure **(G)**.

5.1 Different variations of direct/reverse emulsions

We also tried to vary the emulsion parameters for further improvement of our above results. Details for the optimization attempts included (a) synthesis of MIL-101Cr in reverse emulsion without HNO_3, (b) synthesis of MIL-101Cr in reverse emulsion with NaOH, (c) synthesis of MIL-101Cr in direct emulsion without HNO_3, (d) synthesis of MIL-101Cr in direct emulsion with NaOH, and (e) synthesis of MIL-101Cr in direct emulsion without heptane. As no green MIL-101Cr material could be collected by centrifugation from these synthesis routes we will not discuss the syntheses in detail. Moreover, as no material could be obtained without HNO_3 or the swelling effect of heptane we conclude that both are crucial for the emulsion technique.

5.1.1 Synthesis of MIL-101Cr in direct emulsion without HNO3.

A solution of H_2BDC (0.33 g, 2 mmol), $Cr(NO_3)_3{\cdot}9H_2O$ (0.80 g, 2 mmol), and 5 mL water was combined with a solution of sodium oleate (0,14 g, 0.34 mmol) in 5 mL of water and ultrasonicated with heptane (5 mL) for one minute. The direct emulsion was heated to 180 °C/210 within 30 min and kept at this temperature for 10 min/40 min by microwave heating at 600 W. No green MIL-101Cr material was collected by centrifugation.

5.1.2 Synthesis of MIL-101Cr in direct emulsion with NaOH.

A solution of H_2BDC (0.33 g, 2 mmol), $Cr(NO_3)_3{\cdot}9H_2O$ (0.80 g, 2 mmol), 5 mL water, and 10 µL of 20 % NaOH was combined with a solution of sodium oleate (0,14 g, 0.34 mmol) in 5 mL of water and ultrasonicated with heptane (5 mL) for one minute. The direct emulsion was heated to 180 °C/210 within 30 min and kept at this temperature for 10 min/40 min by microwave heating at 600 W. No green MIL-101Cr material was collected by centrifugation.

5.1.3 Synthesis of MIL-101Cr in direct emulsion with HNO3 but without heptane.

A solution of H_2BDC (0.33 g, 2 mmol), $Cr(NO_3)_3{\cdot}9H_2O$ (0.80 g, 2 mmol), 5 mL water, and 10 µL of 65 % HNO_3 was combined with a solution of sodium oleate (0,14 g, 0.34 mmol) in 10 mL of water and ultrasonicated for one minute. The direct emulsion was heated to 180 °C/210 within 30 min and kept at this temperature for 10 min/40 min by microwave heating at 600 W. No 3green MIL-101Cr material was collected by centrifugation.

5.1.4 Synthesis of MIL-101Cr in reverse emulsion without HNO3.

A solution of H_2BDC (0.33 g, 2 mmol), $Cr(NO_3)_3{\cdot}9H_2O$ (0.80 g, 2 mmol), and 4 mL water was combined with a solution of one of the surfactants, sodium oleate (1.00 g, 3.28 mmol), CTAB (1.00 g, 2.73 mmol), or Triton X-45 (1.00 g, 2.47 mmol) in 4 mL of water, ultrasonicated with *n*-heptane (40 mL) for 1 min, and heated as mentioned above. No green MIL-101Cr material was collected by centrifugation.

5.1.5 Synthesis of MIL-101Cr in reverse emulsion with NaOH.

A solution of H_2BDC (0.33 g, 2 mmol), $Cr(NO_3)_3{\cdot}9H_2O$ (0.80 g, 2 mmol), 4 mL water, and 10 µL of 20 % NaOH was combined with a solution of one of the surfactants, sodium oleate (1.00 g, 3.28 mmol), CTAB (1.00 g, 2.73 mmol), or Triton X-45 (1.00 g, 2.47 mmol) in 4 mL of water and ultrasonicated with *n*-heptane (40 mL) for 1 min. The reverse emulsion was heated to 180 °C within 30 min and kept at this temperatur for 40 min by microwave heating at 600 W (Mars-6 microwave reactor from CEM). No green MIL-101Cr material was collected by centrifugation.

6 Additional analysis for nano/microsized and microMIL-101Cr

6.1 Fourier transform infrared-attenuated total reflectance spectroscopy (FTIR-ATR)

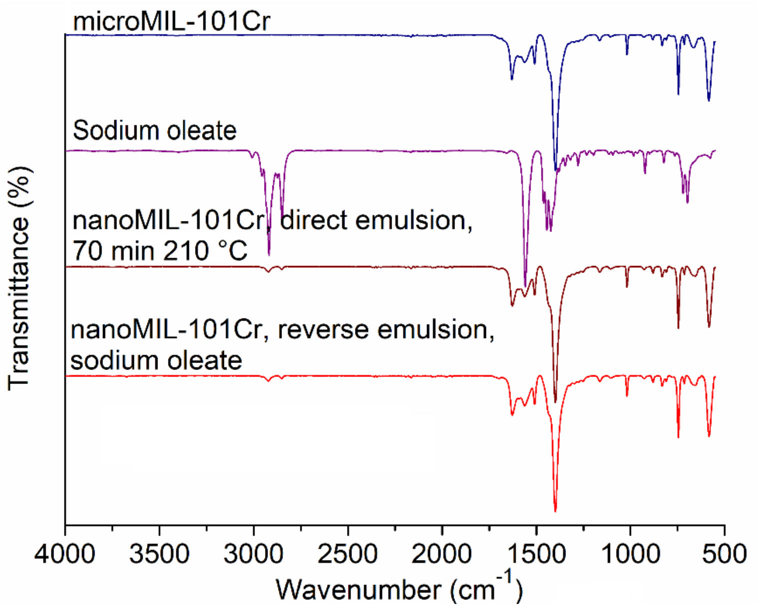

Figure S7 FTIR-ATR spectra of micrMIL-101 (navy blue), sodium oleate (dark violet), nanoMIL-101Cr via direct emulsion (brown) and nanoMIL-101Cr via reverse emulsion (red).

6.2 Pore-size distribution

The following data (**Figure S8 – Figure S10**) show the results for the pore-size distribution of nanoMIL-101Cr via direct and reverse emulsion, and microMIL-101Cr synthesized via a conventional procedure, respectively.

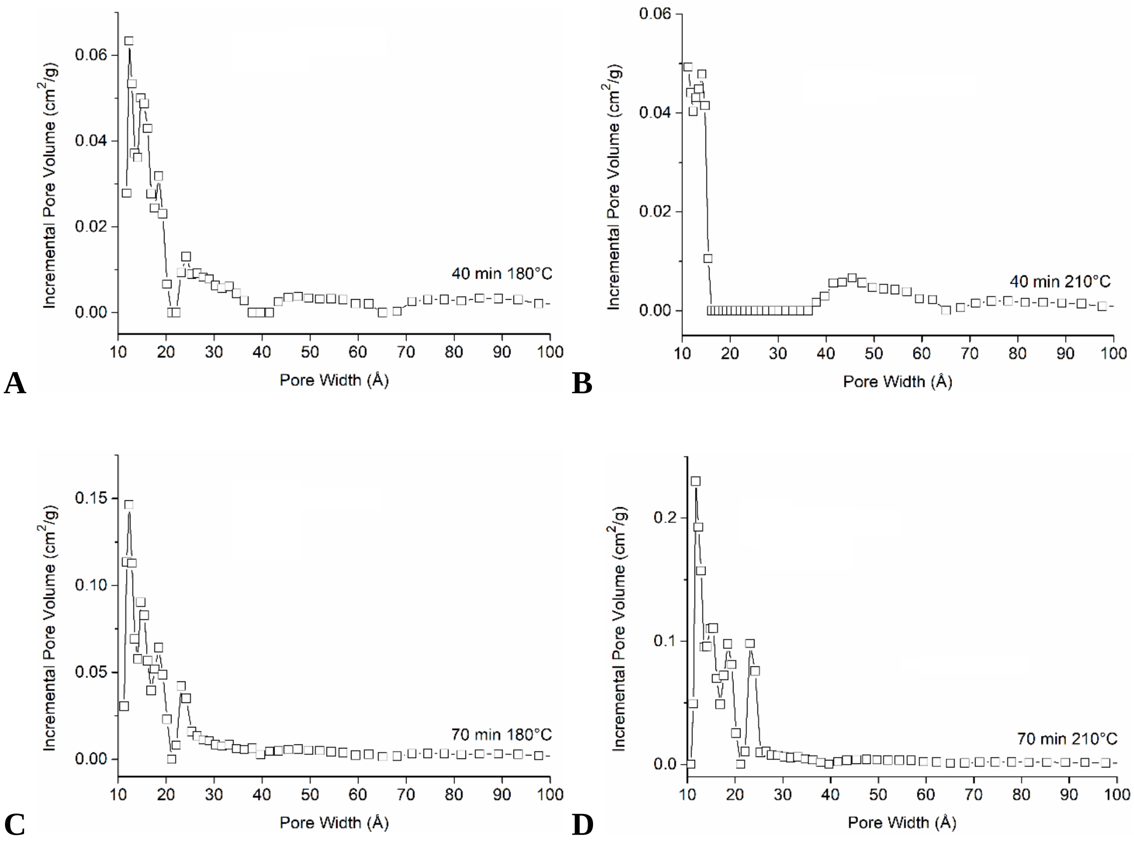

Figure S8 NL-DFT pore-size distribution of nanoMIL-101Cr in direct emulsions prepared for 40 min at 180 °C **(A)**, 70 min at 180 °C **(B)**, 40 min at 210 °C **(C)**, and 70 min at 210 °C **(D)**.

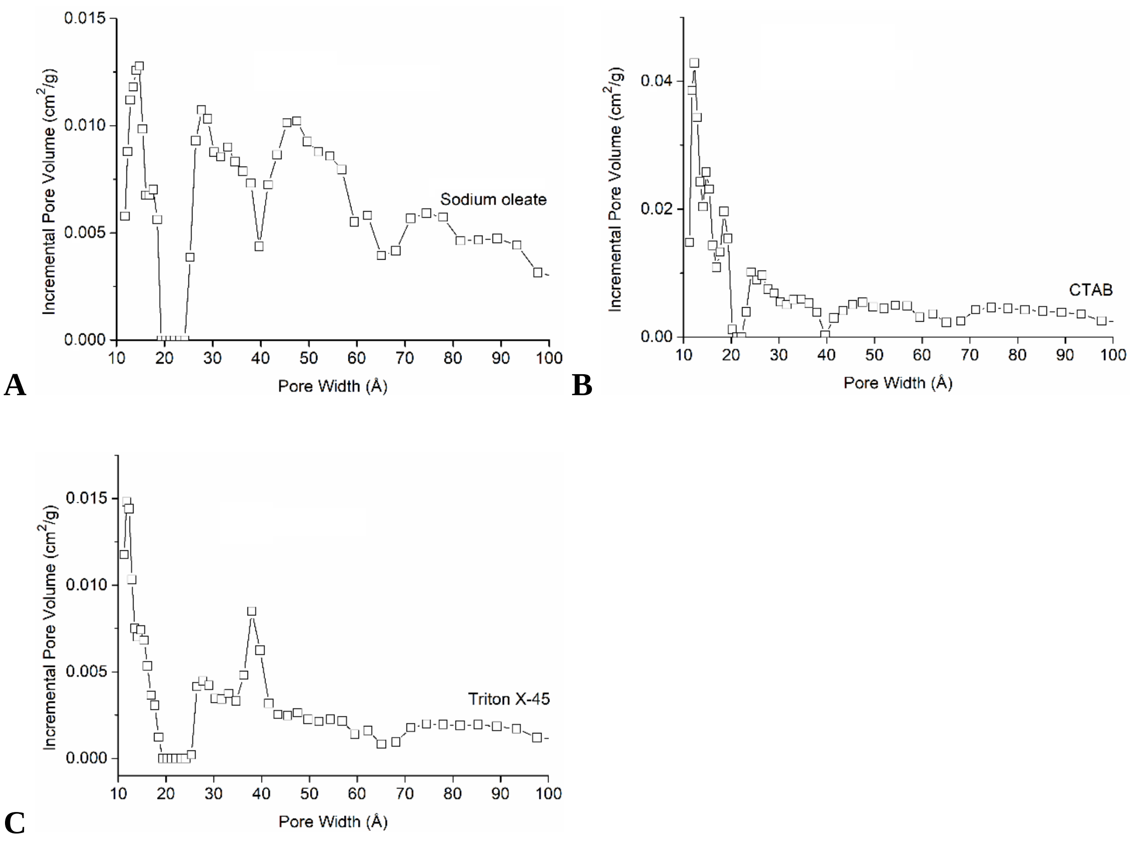

Figure S9 DFT pore-size distribution of nanoMIL-101Cr in reverse emulsions prepared by using sodium oleate **(A)**, CTAB **(B)**, and Triton X-45 **(C)**.

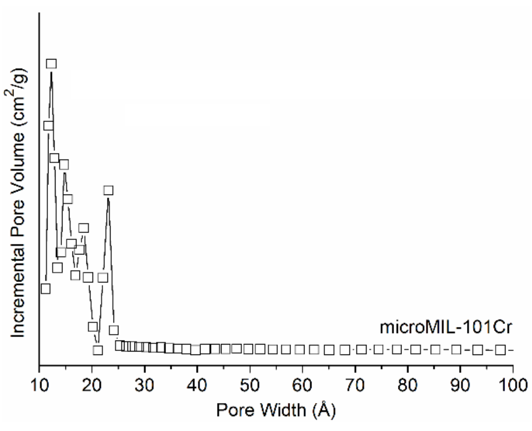

Figure S10 DFT pore-size distribution curves of microMIL-101Cr through conventional synthesis with TMAOH.

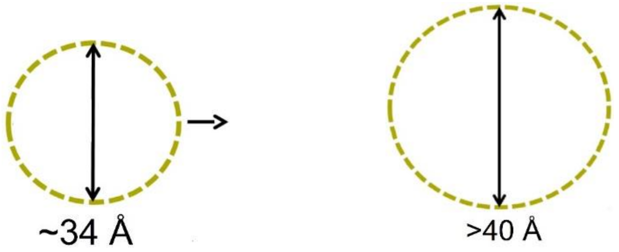

A **B**

Figure S11) The comparison between the normal pore size **(A)** and the pore size given here by using surfactants **(B)** for MIL-101(Cr.

6.3 Scanning electron microscopic (SEM) and transmission electron microscopy (TEM)

Scanning and transmission electron microscopy (SEM, TEM) images show aggregates of small primary particles which may be due to sample preparation, see **Figure 5** and **Figure S12 – Figure S16**. The following data show the images and histograms gotten from TEM measurements of nanoMIL 101Cr samples synthesized via direct and reverse emulsion.

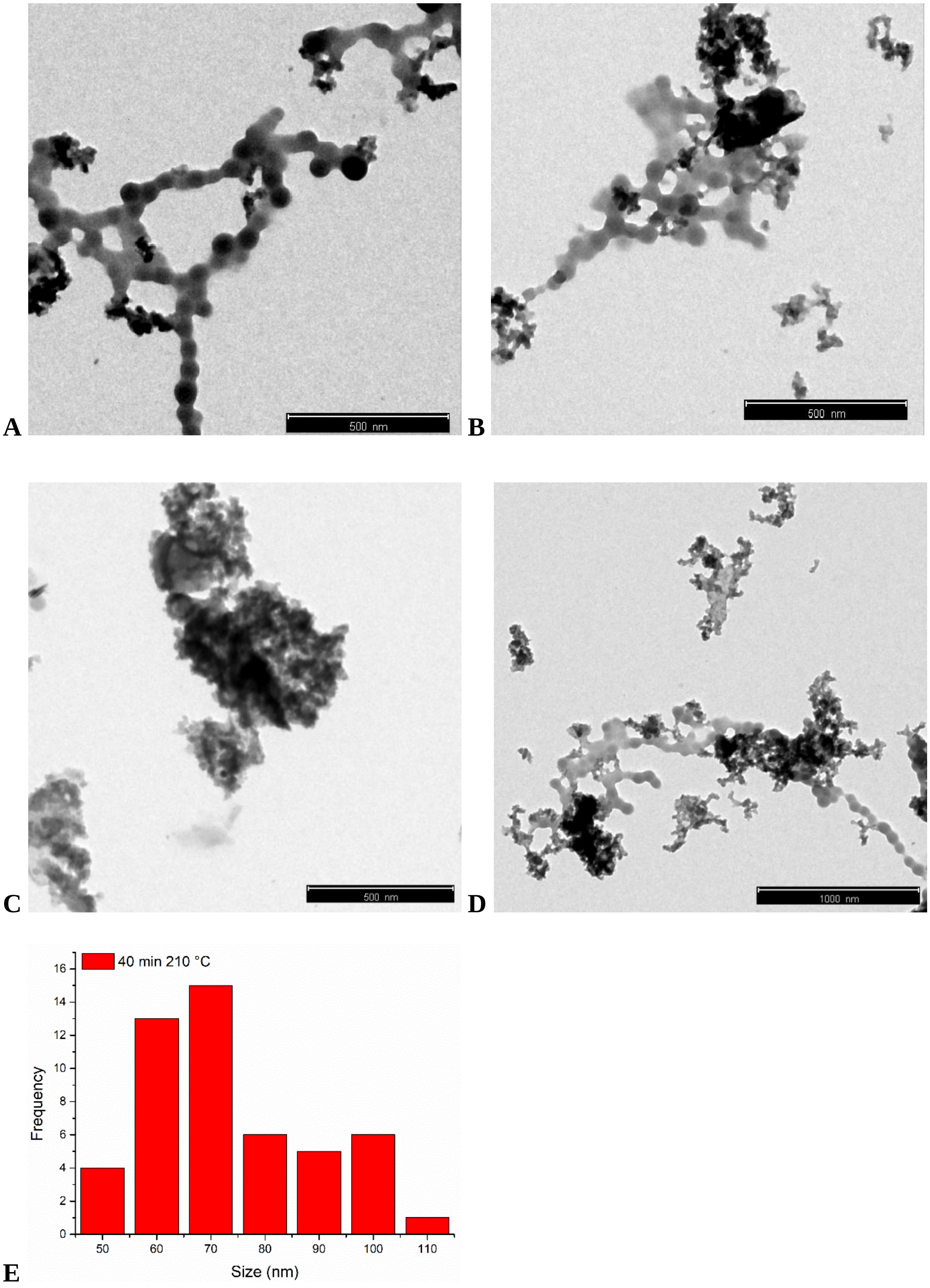

Figure S12 TEM images of nanoMIL-101Cr synthesized in direct emulsion with sodium oleate for 40 minutes at 210° C. Scale bar 500 nm (A-C) and 1000 nm (D). Histogram of nanoMIL-101Cr (E). Statistic based on 50 particles. Polydispersity index (PDI) = 0.08.

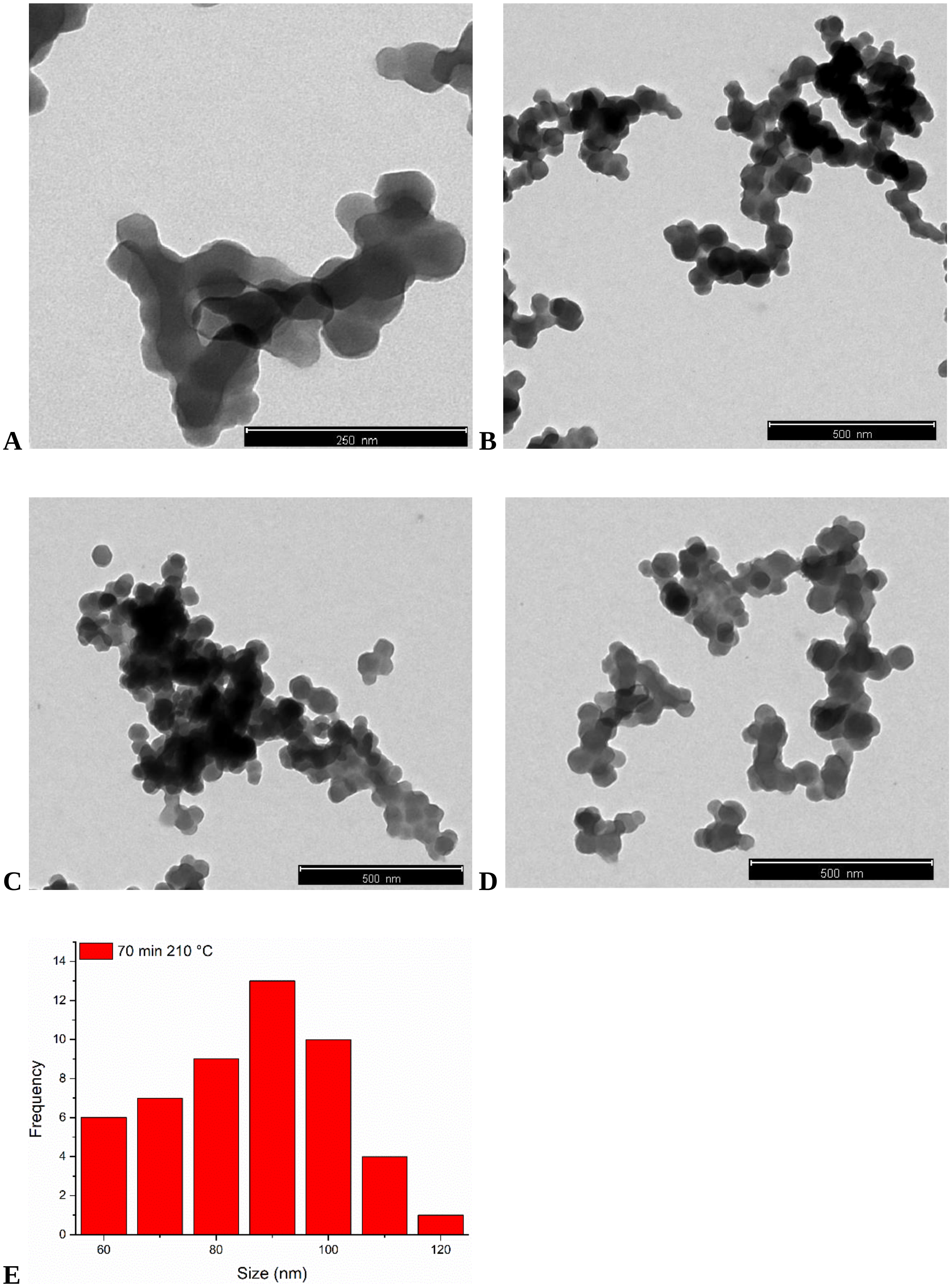

Figure S13 TEM images of nanoMIL-101Cr synthesized in direct emulsion with sodium oleate for 70 minutes at 210° C **(A)**. Scale bar 250 nm **(A)**, 500 nm **(B-D)**. Histogram of nanoMIL-101Cr **(E)**. Statistic based on 50 particles. Polydispersity index (PDI) = 0.05.

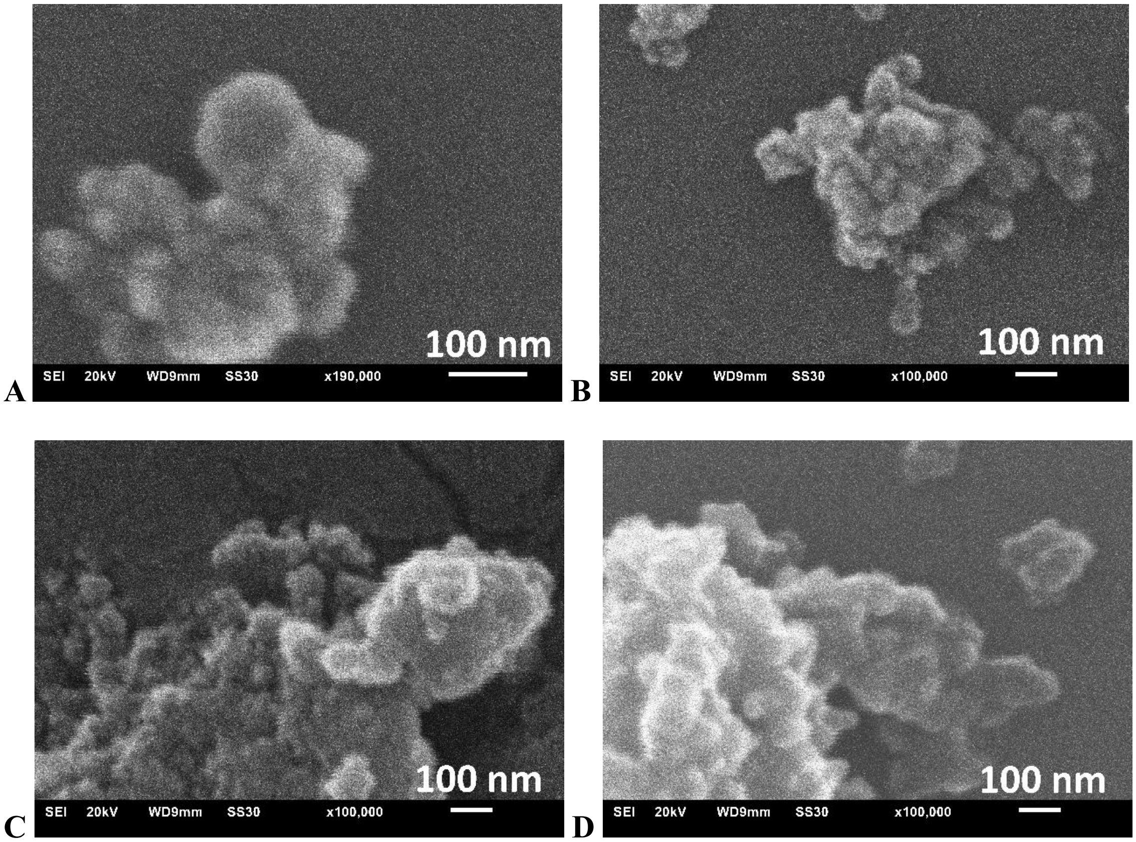

Figure S14 SEM images of the nanoMIL-101Cr prepared in reverse emulsion with sodium oleate **(A**,**B)**; CTAB **(C)**, and Triton X-45 **(D)**.

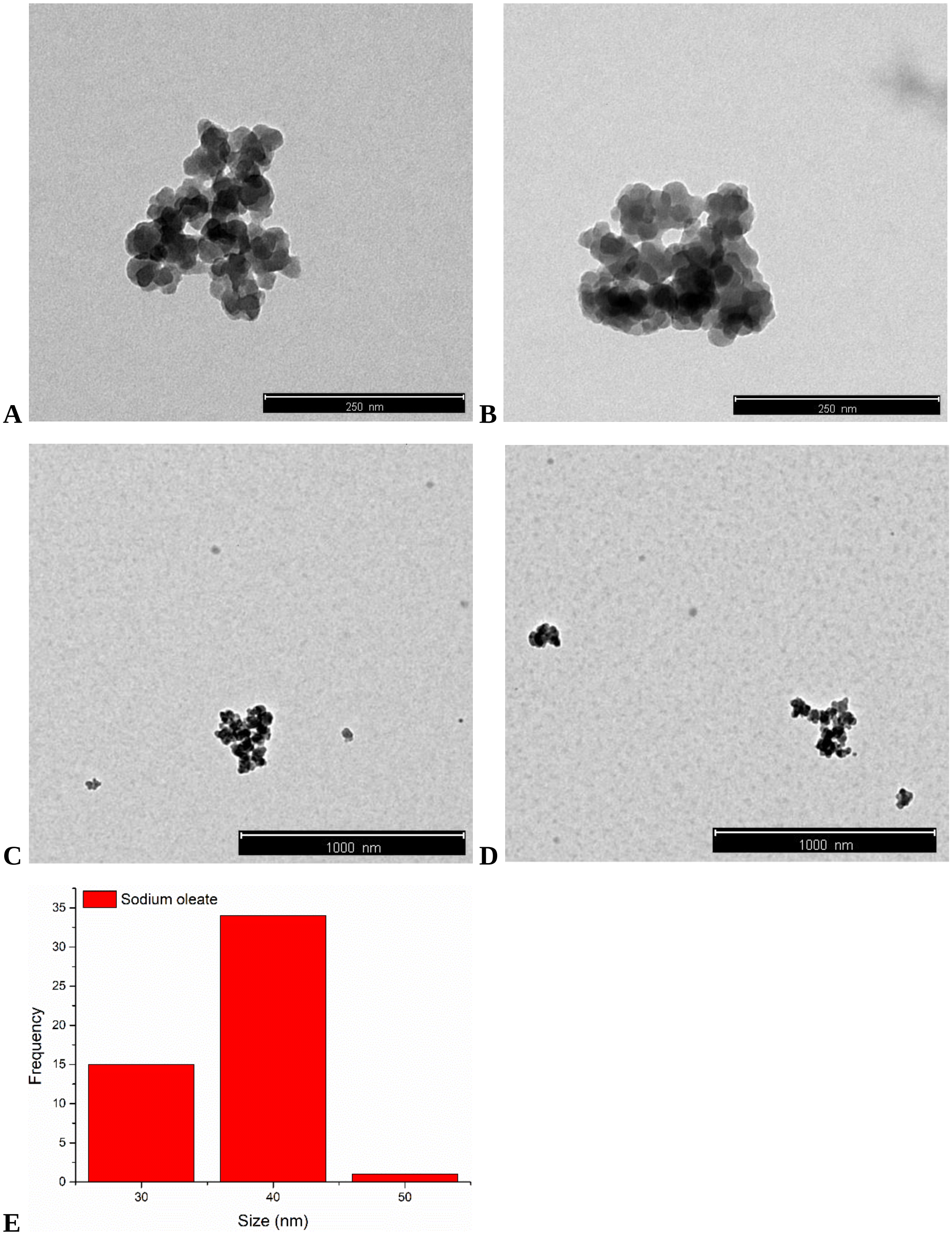

Figure S15 TEM images of nanoMIL-101Cr synthesized in reverse emulsion with sodium oleate. Scale bar 250 nm **(A**,**B)** and 1000 nm **(C,D)**. Histogram of nanoMIL-101Cr **(E)**. Statistic based on 50 particles. Polydispersity index (PDI) = 0.06.

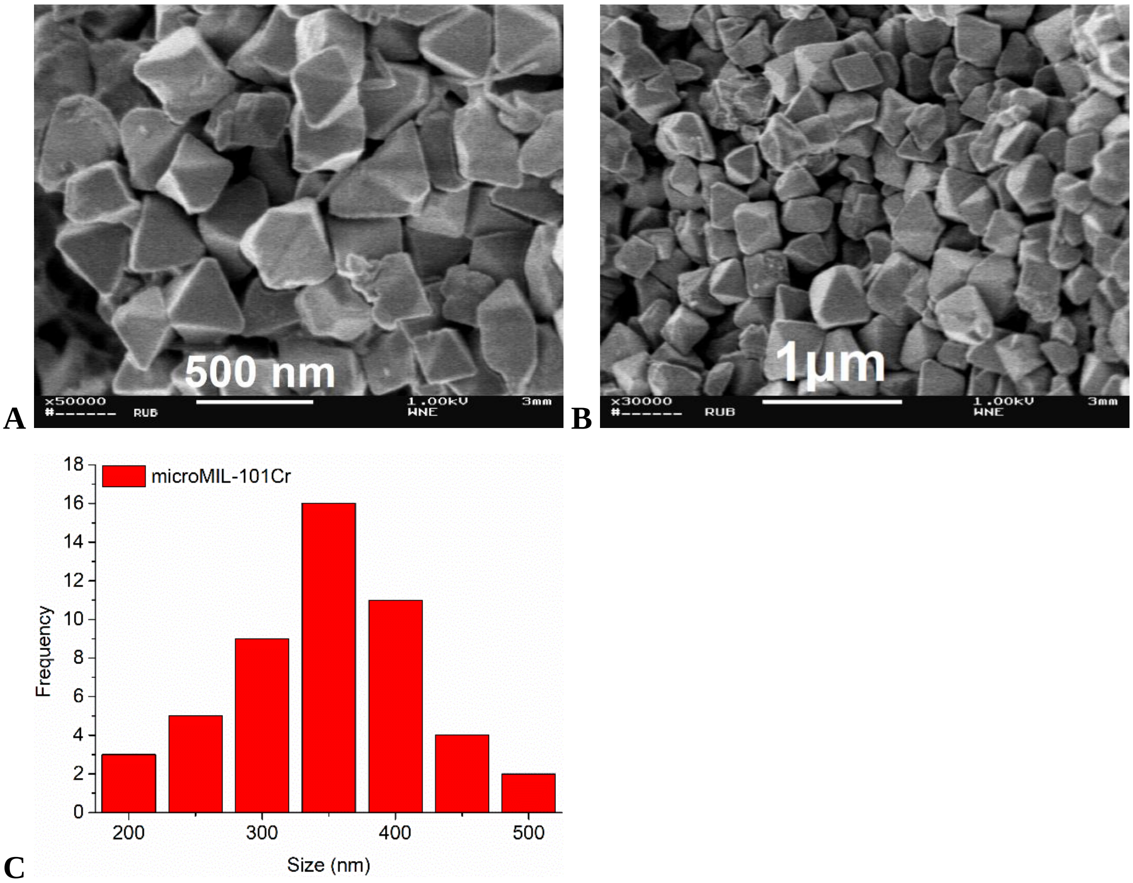

A B C

Figure S16 SEM images for microMIL-101Cr **(A,B)**. Histogram of microMIL-101Cr **(C)**. Statistic based on 50 particles. Polydispersity index (PDI) = 0.04.

6.4 Dynamic light scattering (DLS)

The following figures (**Figure S17 – Figure S23**) show representative intensity-time autocorrelation functions recorded at $\theta = 90°$ **(A)**,the corresponding hydrodynamic radius distribution functions calculated from the relaxation rate distribution functions $G(\Gamma)$ obtained from CONTIN analysis **(B)** as well as the plots of the mean relaxation rate Γ of the dominating scattering species as a function of q^2 **(C)** from DLS measurements of nanoMIL-101Cr samples synthesized via direct and reverse emulsion. In general, we measure typical intensity-time autocorrelation functions that decay to zero at large decay times for all samples. The CONTIN fits (black solid lines) nicely describe each function. The intensity-weighted radius distribution functions obtained from the CONTIN analysis reveal mostly two contributions: 1) a dominating contribution with sizes in the range of hundreds of several hundreds of nm and 2) a contribution at significantly larger radii, in the micron range. In some cases (**Figure S22** and **Figure S23**), also a third contribution close to 100 nm is observed. Thus, the correlation functions are clearly multimodal with two or three contributions. The contributions in the micron size range is most likely caused by the presence of a small fraction of aggregates or impurities from e.g. dust that where not removed by or have formed after the applied filtration process. However, this contribution is only a minor one for each sample and given the fact that the presented results come from an intensity-weighted analysis, can be neglected. Thus, we focus in the following on analysis of the dominating contribution and use angular dependent analysis to study the diffusion behavior and finally determine

the hydrodynamic particle size. Therefore, the mean relaxation rates Γ for the dominating species as obtained from the CONTIN analysis were plotted against the squared scattering vector q^2. For all samples a linear dependence of Γ as a function of q^2 is observed. Thus, we can conclude that we probe only translational diffusion (see equation S10) for all nanoMIL-101Cr samples. The respective translational diffusion coefficients D_t obtained from the slope of the linear regressions in each plot are mentioned in each figure caption, respectively. From D_t we also calculated the respective hydrodynamic radii according to equation S11. The corresponding radii are mentioned in the figure captions. For samples prepared in direct and reverse emulsions with sodium oleate as the surfactant the obtained R_H lie in a range 120-150 nm. For MIL101-Cr samples prepared with CTAB and Triton X-45 as the surfactant values of 726 nm and 440 nm, respectively (**Figure S22** and **Figure S23**) were obtained.

We note that, even small traces of agglomerated particles are able to distort a DLS measurement (Hall et al., 2007). It was shown by Grobelny et. al that the detection of smaller nanoparticles in the presence of a few percent of larger ones by DLS is difficult. Moreover, particles with the size of 10 nm could appear four times as large (~40 nm) while, for example, particles of 80 nm could appear only 1.3-times larger than the particle size by TEM analysis (Tomaszewska et al., 2013). As a consequence, it was not possible to detect the signal coming from 95% of smaller particles in the presence of 5% bigger particles. Other publications also mentioned DLS particle sizes 1.7-times larger than the primary particle size by TEM analysis (Foldbjerg et al., 2009; Chalati et al., 2011). In summary, DLS is very sensitive to traces of larger or agglomerated particles and the detection of smaller particles is easily concealed by a small percentage of agglomerated particles.

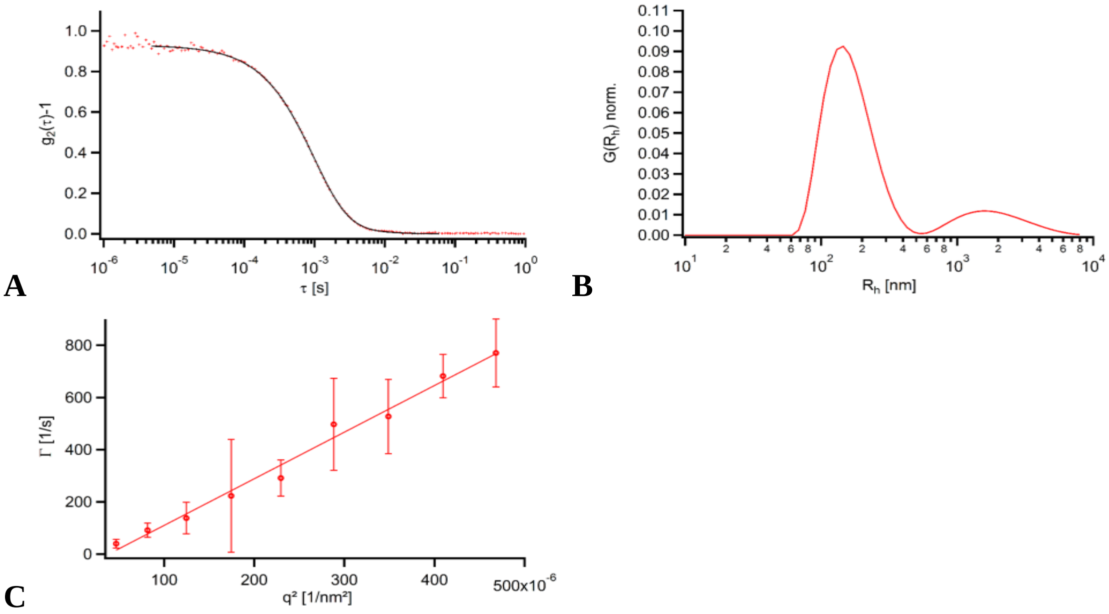

Figure S17 DLS measurement of MIL-101Cr prepared by direct emulsion technique and using sodium oleate as surfactant (40 min, 180 °C). **(A)** Autocorrelation function recorded at 25 °C and a scattering angle of θ = 90 °, **(B)** corresponding hydrodynamic radius distribution function calculated from the relaxation rate distribution function (intensity-weighted). **(C)** Γ vs q^2, the slope of the regression line provides D_t. R_h is calculated using the Stokes-Einstein equation. R_h = 137.25 nm, with $\eta(H_2O)$ = 0.889 mPa*s and $D_t = 1.7861*10^6$ nm²/s.

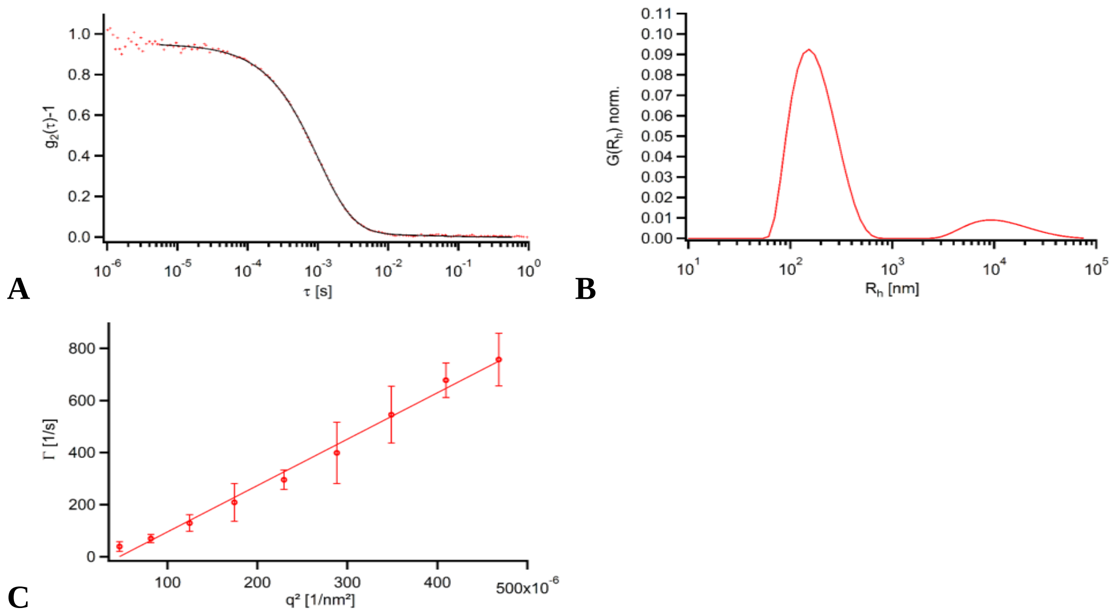

Figure S18 DLS measurement of MIL-101Cr prepared by direct emulsion technique and using sodium oleate as surfactant (70 min, 180 °C). **(A)** Autocorrelation function recorded at 25 °C and a scattering angle of θ = 90 °, ***(B)*** corresponding hydrodynamic radius distribution function calculated from the relaxation rate distribution function (intensity-weighted). **(C)** Γ vs q^2, the slope of the regression line provides D_t. R_h is calculated using the Stokes-Einstein equation. R_h = 137.61 nm, with η(H_2O) = 0.889 mPa*s and $D_t = 1.7814*10^6$ nm²/s.

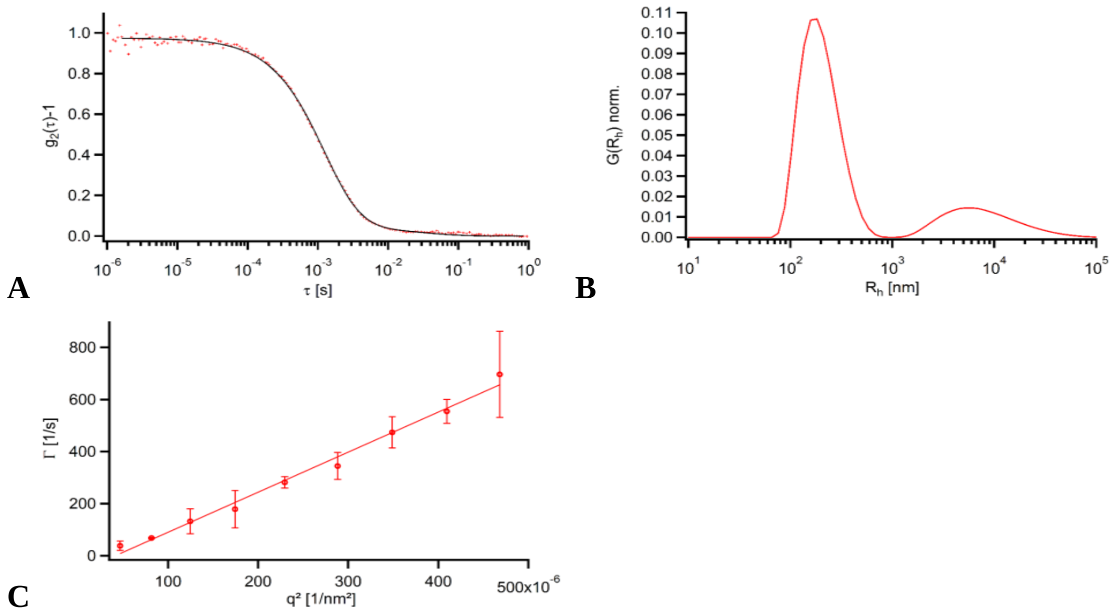

Figure S19 DLS measurement of MIL-101Cr prepared by direct emulsion technique and using sodium oleate as surfactant (40 min, 210 °C). **(A)** Autocorrelation function recorded at 25 °C and a scattering angle of θ = 90 °, **(B)** corresponding hydrodynamic radius distribution function calculated from the relaxation rate distribution function (intensity-weighted). **(C)** Γ vs q^2, the slope of the regression line provides D_t. R_h is calculated using the Stokes-Einstein equation. R_h = 155.04 nm, with η(H_2O) = 0.889 mPa*s and $D_t = 1.5811*10^6$ nm²/s.

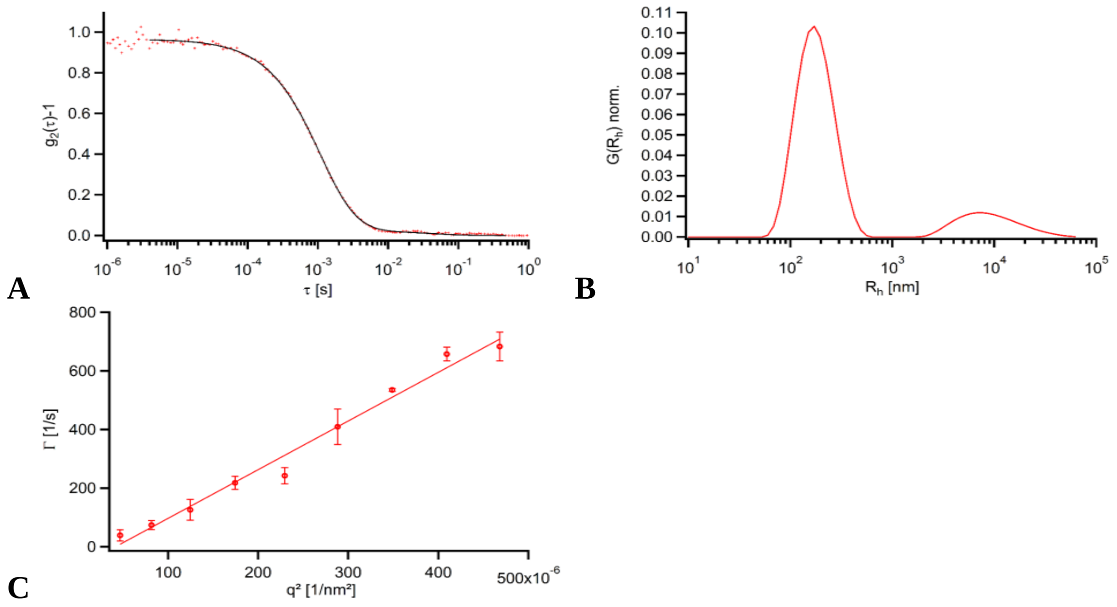

Figure S20 DLS measurement of MIL-101Cr prepared by direct emulsion technique and using sodium oleate as surfactant (70 min, 210 °C). **(A)** Autocorrelation function recorded at 25 °C and a scattering angle of θ = 90 °, **(B)** corresponding hydrodynamic radius distribution function calculated from the relaxation rate distribution function (intensity-weighted). **(C)** Γ vs q^2, the slope of the regression line provides D_t. R_h is calculated using the Stokes-Einstein equation. R_h = 145.36 nm, with $\eta(H_2O)$ = 0.889 mPa*s and $D_t = 1.6853*10^6$ nm²/s.

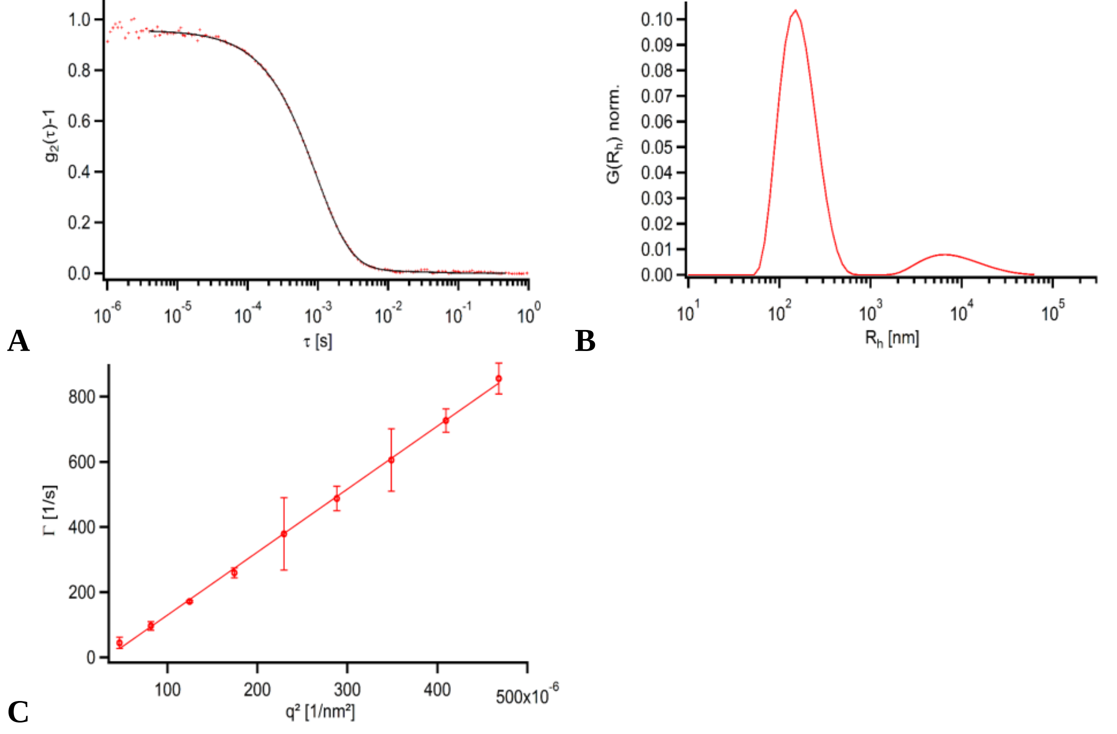

Figure S21 DLS measurement of MIL-101Cr prepared by reverse emulsion technique and using sodium oleate as surfactant. **(A)** Autocorrelation function recorded at 25 °C and a scattering angle of θ = 90 °, **(B)** corresponding hydrodynamic radius distribution function calculated from the relaxation rate distribution function (intensity-weighted. **(C)** Γ vs q^2, the slope of the regression line provides D_t. R_h is calculated using the Stokes-Einstein equation. R_h = 126.86 nm, with $\eta(H_2O)$ = 0.889 mPa*s and $D_t = 1.9323*10^6$ nm²/s.

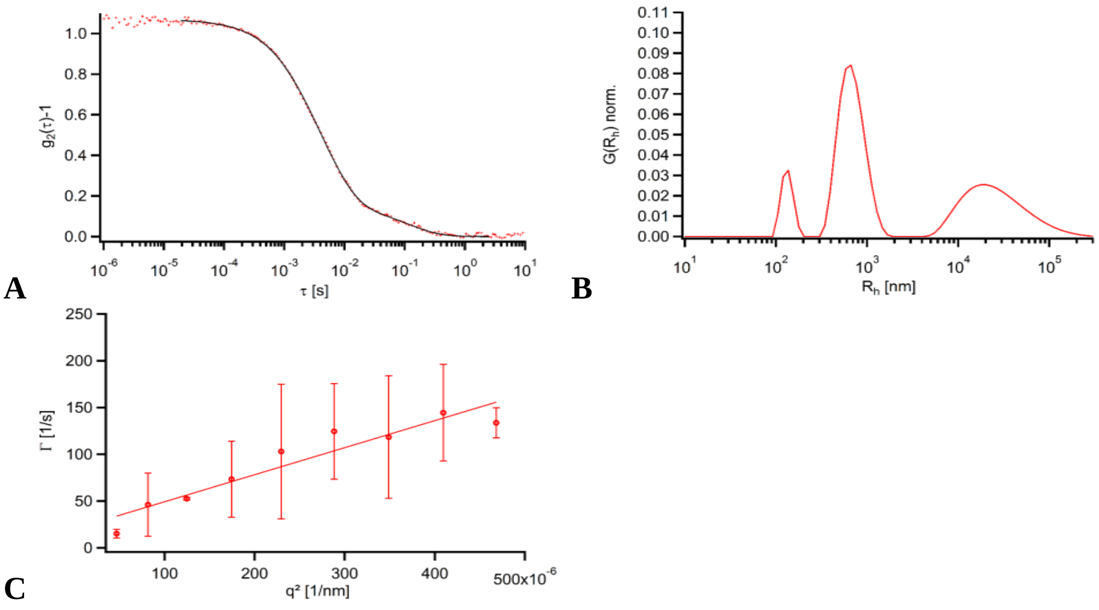

Figure S22 DLS measurement of MIL-101Cr prepared by reverse emulsion technique and using CTAB as surfactant. **(A)** Autocorrelation function recorded at 25 °C and a scattering angle of θ = 90 °, **(B)** corresponding hydrodynamic radius distribution function calculated from the relaxation rate distribution function (intensity-weighted). **(C)** Γ vs q², the slope of the regression line provides D_t. R_h is calculated using the Stokes-Einstein equation. R_h = 726.90 nm, with η(H_2O) = 0.889 mPa*s and D_t = 3.3724*10^5 nm²/s.

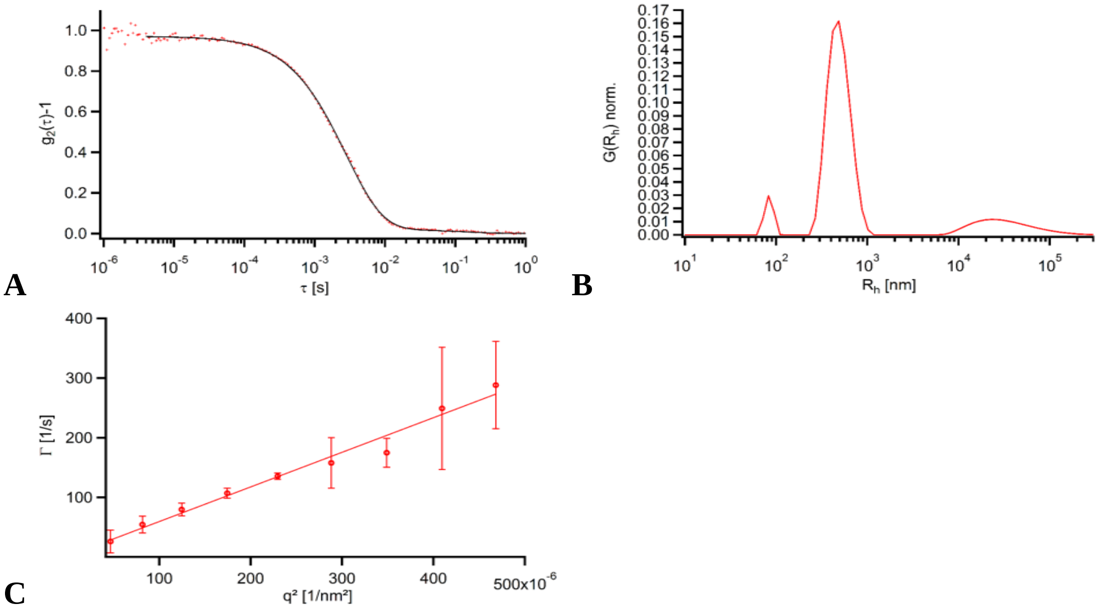

Figure S23 DLS measurement of MIL-101Cr prepared by reverse emulsion technique and using Triton X-45 as surfactant. **(A)** Autocorrelation function recorded at 25 °C and a scattering angle of θ = 90 °, **(B)** corresponding hydrodynamic radius distribution function calculated from the relaxation rate distribution function (intensity-weighted). **(C)** Γ vs q², the slope of the regression line provides D_t. R_h is calculated using the Stokes-Einstein equation. R_h = 441.31 nm, with η(H_2O) = 0.889 mPa*s and D_t = 5.5548*10^5 nm²/s.

7 Mixed-matrix membranes

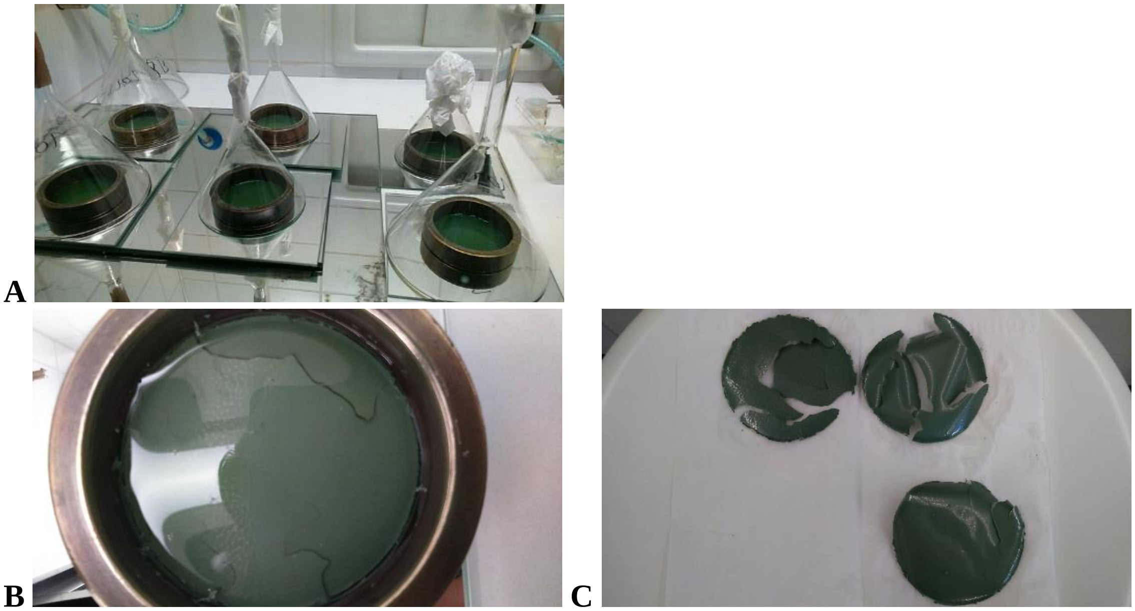

Figure S24 Metal rings with MMM suspension covered with funnels **(A)**. Cracked MMM with 24 wt% of MIL-101Cr **(B**,**C)**.

8 Additional analyses for mixed-matrix membranes

8.1 Powder X-ray diffraction, scanning electron microscopic (SEM), and infrared analysis (IR)

Detail information for the fabrication of Matrimid membranes and mixed-matrix membranes are listed in **Table 2**. Separation properties are summarized in **Table 3**. The results for the PXRD and FTIR-ATR gotten from the MMMs are shown in **Figure S27**. Also, a cross-section of the pure Matrimid membrane is presented in **Figure S25**. Moreover, gas permeation data for CO_2/CH_4 of already published MMMs with different MOFs and Matrimid are listed in **Table S2**.

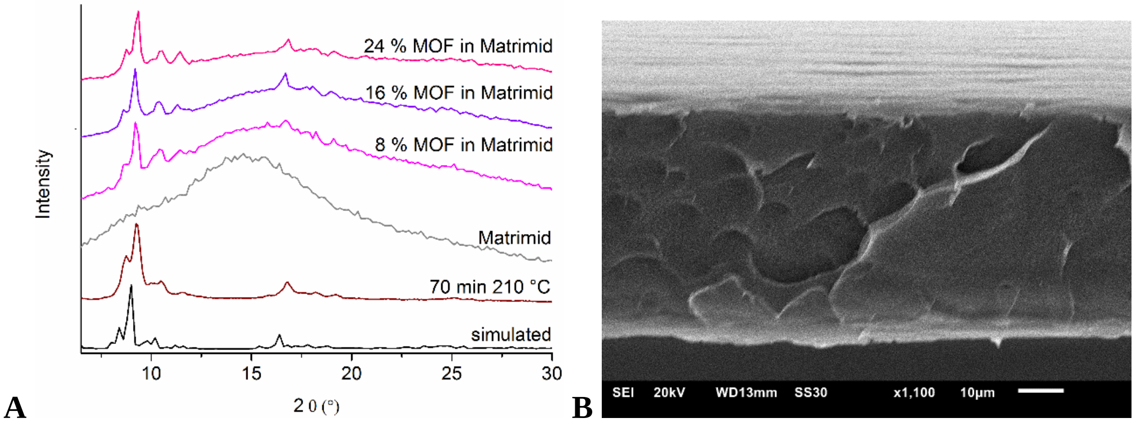

Figure S25 XRD patterns **(A)** of synthesized nanoMIL-101Cr (brown), Matrimid (grey), MMM with 8 wt % MOF (pink); with 16 wt % MOF (violet), with 24 wt % MOF (magenta), simulated pattern (black) (Férey et al., 2005). SEM images of the cross-section of Matrimid membrane **(B)**. Scale bar 10 µm.

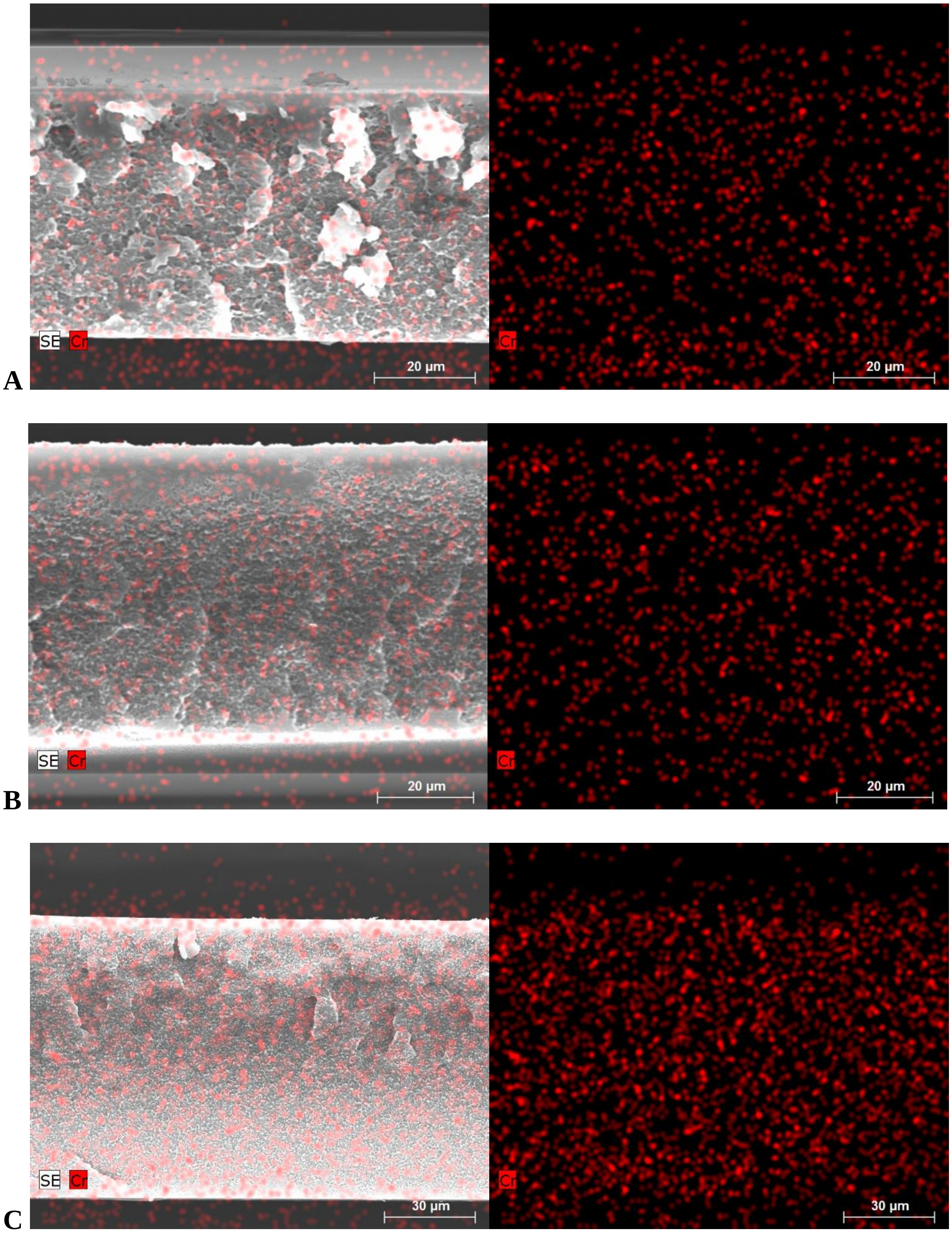

Figure S26 SEM images of the cross-section of microMIL-101Cr@Matrimid membrane: with 8 wt % MOF **(A)**; with 16 wt % MOF **(B)**, and with 20 wt % MOF **(C)**. Images of only EDX mapping (Cr = red) are shown on the left for a clearer representation. The bottom of the cross-sections in the image corresponds to the bottom of the membrane when casted. Cross-section of pure Matrimid is shown in **Figure S25B**.

The FTIR-ATR results for MIL-101Cr, nanoMIL-101Cr, Matrimid, and MMMs show that the characteristic bonds match with those described in the literature (**Figure S27**) (Rajati et al., 2018). The symmetric and asymmetric stretching bonds of the C=O groups of the imide can be observed at 1780 cm^{-1} and 1717 cm^{-1}. The symmetric stretch vibration of benzophenone carbonyl is shown at 1669 cm^{-1}. The bands at 1363 cm^{-1} and 1089 cm^{-1} are for the stretching modes of the C-N-C of the imide 5-membered ring (Inagaki et al., 2013). The band at 1396 cm^{-1} is based on the symmetric O-C-O vibrations of organic linker in the MOF. The bands observed between 1294 cm^{-1} and 1089 cm^{-1} are matching Matrimid bands Inagaki et al. showed previously (Inagaki et al., 2013). The bands at 1366 and 1089 cm^{-1} are for the stretching modes of the C-N-C of the imide 5-membered ring. Between 2800 cm^{-1} and 3000 cm^{-1} comprises C-H stretching including symmetric and asymmetric stretching of CH_2 and CH_3 entities. The vinylic =C-H band can be seen above 3000 cm^{-1}. The carboxylate asymmetric and the symmetric stretching frequencies are located at about 1550 cm^{-1} and 1420 cm^{-1}, respectively. Within this region also bands due to the symmetric and asymmetric bending of methylene and methyl groups appear (Gong et al., 1992).

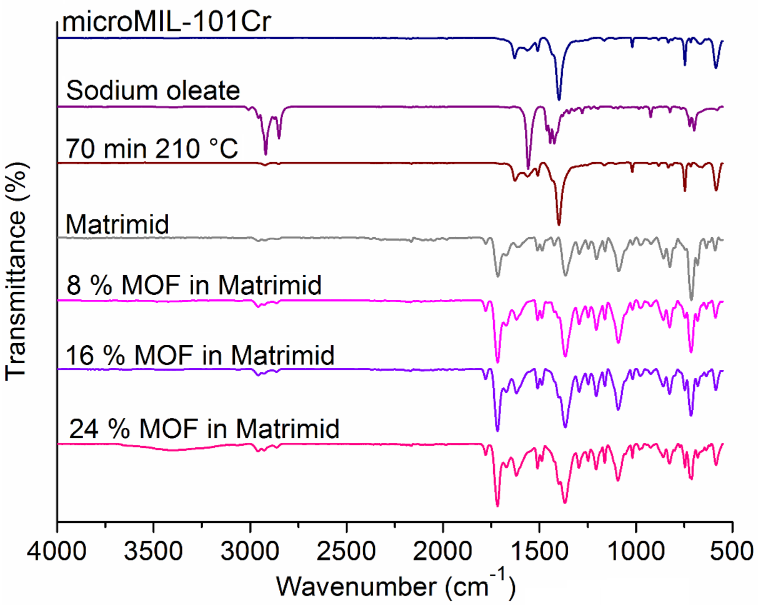

Figure S27 FTIR-ATR spectra of microMIL-101 (navy blue), sodium oleate (dark violet), nanoMIL-101Cr (brown), Matrimid (grey), MMM with 8 wt % MOF (pink); with 16 wt % MOF (violet), with 24 wt % MOF (magenta).

8.2 Maxwell and Bruggeman model

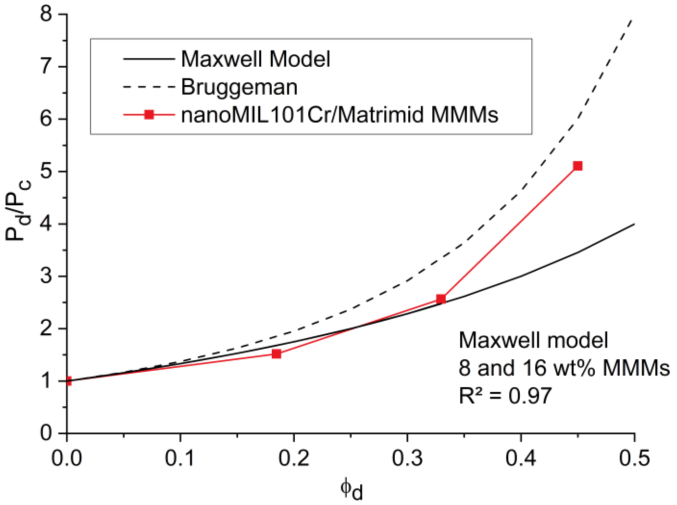

Figure S28 Relative experimental CH_4 permeabilities (referenced to the permeability P_c of the pure polymer membrane) for nanoMIL-101Cr-101Cr@Matrimid (red curve) with different filler volume fraction ϕ_d. The black solid line gives the theoretical CH_4 permeabilities for porous fillers based on the Maxwell Model and the black dashed line gives the theoretical CH_4 permeabilities for porous fillers based on the Bruggeman Model.

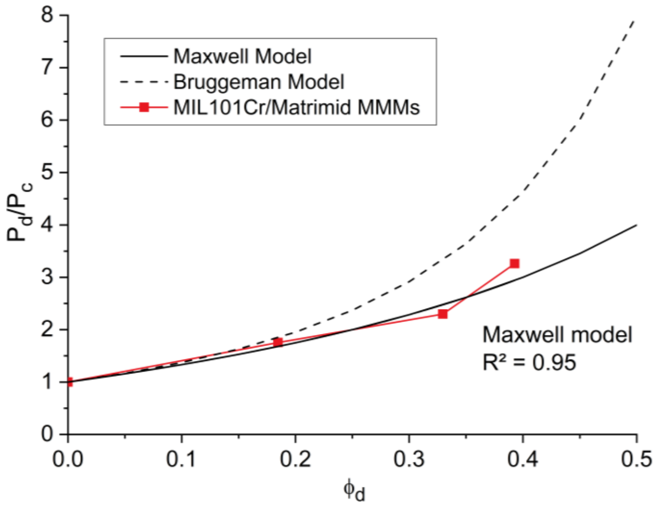

Figure S29 Relative experimental CO_2 permeabilities (referenced to the permeability P_c of the pure polymer membrane) for microMIL-101Cr@Matrimid (red curve) with different filler volume fraction ϕ_d. The black solid line gives the theoretical CO_2 permeabilities for porous fillers based on the Maxwell Model and the black dashed line gives the theoretical CO_2 permeabilities for porous fillers based on the Bruggeman Model.

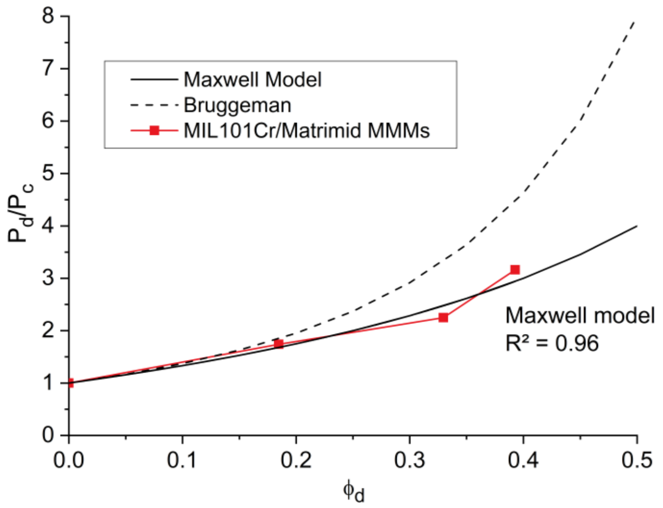

Figure S30 Relative experimental CH_4 permeabilities (referenced to the permeability Pc of the pure polymer membrane) for microMIL-101Cr@Matrimid (red curve) with different filler volume fraction ϕd. The black solid line gives the theoretical CH_4 permeabilities for porous fillers based on the Maxwell Model and the black dashed line gives the theoretical CH_4 permeabilities for porous fillers based on the Bruggeman Model.

9 References

Anjum, M.W., Bueken, B., De Vos, D., Vankelecom, I.F. (2016). MIL-125(Ti) based mixed matrix membranes for CO_2 separation from CH_4 and N_2. J. Membr. Sci. 502, 21-28. doi: 10.1016/j.memsci.2015.12.022

Chalati, T., Horcajada, P., Gref, R., Couvreur, P., Serre, C. (2011). Optimisation of the synthesis of MOF nanoparticles made of flexible porous iron fumarate MIL-88A. J. Mater. Chem. 21, 2220-2227. doi: 10.1039/C0JM03563G

Dorosti, F., Omidkhah, M., Abedini, R. (2014). Fabrication and characterization of Matrimid/MIL-53 mixed matrix membrane for CO 2 /CH 4 separation. Chem. Eng. Res. Des. 92, 2439-2448. doi: 10.1016/j.cherd.2014.02.018

Férey, G., Draznieks, C.M., Serre, C., Millange, F., Dutour, J., Surble, S., Margiolaki, I. (2005). A Chromium Terephthalate-Based Solid with Unusually Large Pore Volumes and Surface Area. Science 309, 2040-2042. doi: 10.1126/science.1116275

Foldbjerg, R., Olesen, P., Hougaard, M., Dang, D.A., Hoffmann, H.J., Autrup, H. (2009). PVP-coated silver nanoparticles and silver ions induce reactive oxygen species, apoptosis and necrosis in THP-1 monocytes. Toxicol. Lett. 190, 156-162. doi: 10.1016/j.toxlet.2009.07.009

Gong, W.Q., Parentich, A., Little, L.H., Warren, L.J. (1992). Adsorption and Immobilization of Cytochrome c on Nanodiamonds. Langmuir 8, 118-124. doi: 10.1021/la0495736

Hall, J.B.; Dobrovolskaia, M.A., Patri, A.K., McNeil, S.E. (2007). Characterization of nanoparticles for therapeutics. Nanomedicine, 2, 789-803. doi: 10.2217/17435889.2.6.789

Herbst, A., and Janiak, C. (2016). Selective glucose conversion to 5-hydroxymethyl-furfural (5-HMF) instead of levulinic acid with MIL-101Cr MOF-derivatives. New J. Chem. 40, 7958-7967. doi: 10.1039/C6NJ01399F

Huang, X.-X., Qiu, L.-G., Zhang, W., Yuan, Y.-P., Jiang, X., Xie, A.-J., Shena, Y.-H., Zhub, J.-F. (2012). Hierarchically mesostructured MIL-101 metal–organic frameworks: supramolecular template-directed synthesis and accelerated adsorption kinetics for dye removal. CrystEngComm 14, 1613-1617. doi: 10.1039/C1CE06138K

Inagaki, M., Ohta, N., Hishiyama, Y. (2013). Aromatic polyimides as carbon precursors. Carbon 61, 1-21. doi: 10.1016/j.carbon.2013.05.035

Jeremias, F., Henninger, S.K., Janiak, C. (2016). Ambient pressure synthesis of MIL-100(Fe) MOF from homogeneous solution using a redox pathway. Dalton Trans. 45 (20), 8637-8644. doi: 10.1039/C6DT01179A

Jiang, D., Burrows, A.D., Edler, K.J. (2011). Size-controlled synthesis of MIL-101(Cr) nanoparticles with enhanced selectivity for CO_2 over N_2. CrystEngComm 12, 6916-6919. doi: 10.1039/C1CE06274C

Khan, N.A., Kang, I.J., Seok, H.Y., Jhung, S.H. (2011). Facile synthesis of nano-sized metal-organic frameworks, chromium-benzenedicarboxylate, MIL101. Chem. Eng. J. 166, 1152-1157. doi: 10.1016/j.cej.2010.11.098

Khutia, A., Rammelberg, H.U., Schmidt, T., Henninger, S., Janiak, C. (2013). Water Sorption Cycle Measurements on Functionalized MIL-101Cr for Heat Transformation Application. Chem. Mater. 25, 790-798. doi: 10.1021/cm304055k

Khutia, A., and Janiak, C. (2014). Programming MIL-101Cr for selective and enhanced CO_2 adsorption at low pressure by postsynthetic amine functionalization. Dalton Trans. 43, 1338-1347. doi: 10.1039/C3DT52365A

Naseri, M., Mousavi, S.F., Mohammadi, T., Bakhtiari, O. (2015). Synthesis and gas transport performance of MIL-101/Matrimid mixed matrix membranes. J. Ind. Eng. Chem. 29, 249-256. doi: 10.1016/j.jiec.2015.04.007

Nik, O.G., Chen, X.Y., Kaliaguine, S. (2012). Functionalized metal organic framework-polyimide mixed matrix membranes for CO_2/CH_4 separation. J. Membr. Sci. 4213, 48-61. doi: 10.1016/j.memsci.2012.04.003

Perez, E.V., Balkus Jr., K.J., Ferraris, J.P., Musselman, I.H. (2009). Mixed-matrix membranes containing MOF-5 for gas separations. J. Membr. Sci. 328, 165-173. doi: 10.1016/j.memsci.2008.12.006

Rajati, H., Navarchian, A.H., Tangestaninejad, S. (2018). Preparation and Characterization of Mixed Matrix Membranes based on Matrimid/PVDF blend and MIL-101(Cr) as filler for CO 2 /CH 4 separation. Chem. Eng. Sci. 185, 92-104. doi: 10.1016/j.ces.2018.04.006

Rezk, A., Al-Dadah, R., Mahmoud, S., Elsayed, A. (2013). Investigation of Ethanol/metal organic frameworks for low temperature adsorption cooling applications. Appl. Energy 112, 1025-1031. doi: 10.1016/j.apenergy.2013.06.041

Shahid, S., and Nijmeijer, K. (2014). High pressure gas separation performance of mixed-matrix polymer membranes containing Highmesoporous FE(BTC). J. Membr. Sci. 459, 33-44. doi: 10.1016/j.memsci.2014.02.009

Shen, T., Luo, J., Zhang, S., Luo, X., Environ. J. (2015). Hierarchically mesostructured MIL-101 metal–organic frameworks with different mineralizing agents for adsorptive removal of methyl orange and methylene blue from aqueous solution. Chem. Eng. 3, 1372-1383. doi: 10.1016/j.jece.2014.12.006

Tanh Jeazet, H.B., Staudt, C., Janiak, C. (2012). A method for increasing permeability in O_2/N_2 separation with mixed-matrixmembranes made of water-stable MIL-101 and polysulfone. Chem. Commun. 48, 2140-2142. doi: 10.1039/C2CC16628C

Tomaszewska, E., Soliwoda, K., Kadziola, K., Tkacz-Szczesna, B., Celichowski, G., Cichomski, M., Szmaja, W., Grobelny, J. (2013). Detection Limits of DLS and UV-Vis Spectroscopy in Characterization of Polydisperse Nanoparticles Colloids. J. Nanomater. 60, 1-10. doi: 10.1155/2013/313081

Wickenheisser, M., Jeremias, F., Henninger, S.K., Janiak, C. (2013). Grafting of hydrophilic ethylene glycols or ethylenediamine on coordinatively unsaturated metal sites in MIL-100(Cr) for improved water adsorption characteristics. Inorg. Chim. Acta 407, 145-152. doi: 10.1016/j.ica.2013.07.024

Wickenheisser, M., and Janiak, C. (2015). Hierarchical embedding of micro-mesoporous MIL-101(Cr) in macroporous poly(2-hydroxyethyl methacrylate) high internal phase emulsions with monolithic shape for vapor adsorption applications. Microporous Mesoporous Mater. 204, 242-250. doi: 10.1016/j.micromeso.2014.11.025

Wuttke, S., Braig, S., Preiß, T., Zimpel, A., Sicklinger, J., Bellomo, C., Rädler, J.O., Vollmar, A.M., Bein, T. (2015). MOF nanoparticles coated by lipid bilayers and their uptake by cancer cells. Chem. Commun. 51, 15752-15755. doi: 10.1039/C5CC06767G

Zhang, Y., Musselman, I.H., Ferraris, J.P., Balkus Jr., K.J. (2008). Gas permeability properties of Matrimid (R) membranes containing the metal-organic framework Cu-BPY-HFS. J. Membr. Sci. 313, 170-181. doi: 10.1016/j.memsci.2008.01.005

Zhao, T., Jeremias, F., Boldog, I., Nguyen, B., Henninger, S.K., Janiak, C. (2015). High-yield, fluoride-free and large-scale synthesis of MIL-101(Cr). Dalton Trans. 44, 16791-16801. doi: 10.1039/C5DT02625C
